# Risk Factors for Indeterminate Interferon-Gamma Release Assay for the Diagnosis of Tuberculosis in Children—A Systematic Review and Meta-Analysis

**DOI:** 10.3389/fped.2019.00208

**Published:** 2019-05-29

**Authors:** Noëmi R. Meier, Thomas Volken, Marc Geiger, Ulrich Heininger, Marc Tebruegge, Nicole Ritz

**Affiliations:** ^1^Mycobacterial Research Laboratory, University of Basel Children's Hospital, Basel, Switzerland; ^2^Faculty of Medicine, University of Basel, Basel, Switzerland; ^3^School of Health Professions, Zürich University of Applied Sciences, Winterthur, Switzerland; ^4^Paediatric Infectious Diseases and Vaccinology Unit, University of Basel Children's Hospital, Basel, Switzerland; ^5^UCL Great Ormond Street Institute of Child Health, University College London, London, United Kingdom; ^6^Department of Paediatric Infectious Diseases and Immunology, Evelina London Children's Hospital, Guy's and St. Thomas' NHS Foundation Trust, London, United Kingdom; ^7^Royal Children's Hospital Melbourne, Department of Paediatrics, University of Melbourne, Melbourne, VIC, Australia

**Keywords:** Clinical studies, IGRA, latent, pediatrics, risk difference, QuantiFERON, T-SPOT.TB, T cell response

## Abstract

**Background:** Interferon-gamma release assays (IGRA) are well-established immunodiagnostic tests for tuberculosis (TB) in adults. In children these tests are associated with higher rates of false-negative and indeterminate results. Age is presumed to be one factor influencing cytokine release and therefore test performance. The aim of this study was to systematically review factors associated with indeterminate IGRA results in pediatric patients.

**Methods:** Systematic literature review guided by the preferred reporting items for systematic reviews and meta-analyses (PRISMA) searching PubMed, EMBASE, and Web of Science. Studies reporting results of at least one commercially available IGRA (QuantiFERON-TB, T-SPOT.TB) in pediatric patient groups were included. Random effects meta-analysis was used to assess proportions of indeterminate IGRA results. Heterogeneity was assessed using the I^2^ value. Risk differences were calculated for studies comparing QuantiFERON-TB and T-SPOT.TB in the same study. Meta-regression was used to further explore the influence of study level variables on heterogeneity.

**Results:** Of 1,293 articles screened, 133 studies were included in the final analysis. These assessed QuantiFERON-TB only in 77.4% (103/133), QuantiFERON-TB and T-SPOT.TB in 15.8% (21/133), and T-SPOT.TB only in 6.8% (9/133) resulting in 155 datasets including 107,418 participants. Overall 4% of IGRA results were indeterminate, and T-SPOT.TB (0.03, 95% CI 0.02–0.05) and QuantiFERON-TB assays (0.05, 95% CI 0.04–0.06) showed similar proportions of indeterminate results; pooled risk difference was−0.01 (95% CI −0.03 to 0.00). Significant differences with lower proportions of indeterminate assays with T-SPOT.TB compared to QuantiFERON-TB were only seen in subgroup analyses of studies performed in Africa and in non-HIV-infected immunocompromised patients. Meta-regression confirmed lower proportions of indeterminate results for T-SPOT.TB compared to QuantiFERON-TB only among studies that reported results from non-HIV-infected immunocompromised patients (*p* < 0.001).

**Conclusion:** On average indeterminate IGRA results occur in 1 in 25 tests performed. Overall, there was no difference in the proportion of indeterminate results between both commercial assays. However, our findings suggest that in patients in Africa and/or patients with immunocompromising conditions other than HIV infection the T-SPOT.TB assay appears to produce fewer indeterminate results.

## Introduction

Tuberculosis (TB) remains the leading cause of mortality by a single infectious agent, accounting for an estimated 1.6 million deaths worldwide. According to the latest report by the World Health Organization 10 million people are estimated to have developed TB disease in 2017 (1). However, the majority of individuals infected with *Mycobacterium tuberculosis* are asymptomatic and remain in a latent stage of infection. Data on infected individuals is not included in the World Health Organization TB report as TB infection is not a notifiable disease. Therefore, only estimates exist with one of the most recent estimates suggesting that in 2014 a total of 1.7 billion individuals, equivalent to 23% of the global population, had latent TB infection (2).

Progression from latent TB infection to active TB disease occurs in approximately one in ten adults. Children, however, progress more frequently to active TB and progression may be particularly rapid in the first 2 years of life (3–5). Early diagnosis and treatment are therefore key to reduce the burden of active TB in children.

Immuno-diagnostic tests are the main tools for the diagnosis of latent TB infection and both the tuberculin skin test (TST) and interferon-gamma release assays (IGRA) are used in the clinical setting (6, 7). The latter have been developed to overcome the limited specificity of the TST (8, 9). In adults the two commercially available IGRA, the QuantiFERON-TB and T-SPOT.TB—both existing in several test generations—have replaced the TST in many settings, primarily in an attempt to improve specificity (10).

In children, there is evidence that IGRA may have limited sensitivity and therefore the TST is still advocated by most experts (11–14). In addition, indeterminate IGRA results—due to either high interferon-γ background concentration in the negative control or low interferon-γ response in the positive control—have been shown to be more frequent in children compared to adults (15–18).

Underlying reasons for higher proportions of indeterminate IGRA results in children are largely speculative, but several contributing factors including age, concomitant infections and malnutrition have been postulated (18–20).

The aim of this study was to summarize the existing data on indeterminate IGRA results in children and determine key influencing variables.

## Methods

### Study Selection

A systematic literature search of studies reporting IGRA results in children was performed using PubMed, Embase, and Web of Science. Studies published until October 1st, 2018 were considered. The study was done according to the preferred reporting items for systematic reviews and meta-analyses (PRISMA) statement (21) (Supplementary Material 1 PRISMA Checklist). The following search terms were used: (tuberculosis OR TB) AND [(((t-spot.tb) OR t-spot) OR quantiferon-tb) OR quantiferon] AND (children OR pediatric OR pediatric). The following inclusion criteria were used (i) patients in the pediatric age range with a mean or median age <18 years and a maximum upper age range of 24 years, (ii) results of at least one of the commercially available IGRA detailed (including a statement about indeterminate test results), (iii) publication in English, French, or German. Case reports, case series, conference abstracts and studies involving fewer than 10 participants, commentaries and reviews were excluded. The search and selection of included studies was done by MG, NM, and NR. In unclear cases a joint decision for inclusion or exclusion of the study was made.

### Data Extraction

Data were extracted using a standard form including the following variables: year of publication, country in which the study was done, number of participants, mean or median age of participants, age range of participants, type of test performed, number of positive, negative and indeterminate results, definition of indeterminate result, Bacillus Calmette-Guérin (BCG) vaccination status, human immunodeficiency virus (HIV) infection status and information on other potential immunocompromising conditions (e.g., rheumatic diseases, cancer) and concomitant infections (e.g., helminth or other parasitic infections).

### Statistical Analysis

The primary outcome was the proportion of indeterminate IGRA results, which was calculated as the number of indeterminate test results divided by the total number of valid test results. Stratified meta-analyses for proportions were performed using a random effects model and the DerSimonian and Laird method, with the estimate of heterogeneity taken from the inverse-variance fixed-effect model. Stratification variables comprised type of IGRA used (QuantiFERON-TB and T-SPOT.TB), age groups (0–7 ≥ 8 years), geographical location of the population under study (Africa, Australia, North America, South America, Asia, Europe) and immune status (HIV infection rate groups, and presence of other immunocompromising factors). Heterogeneity was determined using the I^2^ statistic.

In studies comparing both QuantiFERON-TB and T-SPOT.TB, additional stratified meta-analyses for risk differences were performed. Risk differences were defined as the difference in the proportion of indeterminate results between the two IGRA tests and were calculated according to Newcombe and Altman (22). For comparison of pooled risk difference, we applied the DerSimonian and Laird risk difference method. Study weight was indicated by using random effect models for the individual studies to account for the different study characteristics. For risk difference analysis stratification for age groups were done in two groups (0–7 ≥ 8 years) because of the limited number of available datasets.

To further explore potential sources of heterogeneity, we used meta-regression if I^2^ was higher than 30%. We considered the following variables as potentially explanatory in a multivariable model: type of IGRA used, age group, geographic location of the population under study, immune status (HIV or other immunocompromising conditions) were considered as explanatory variables in a multivariable model.

We used GraphPad Prism Version 7.02 (GraphPad Software, San Diego, CA, USA) and Stata Version 15.1 (StataCorp, College Station, TX, USA) to generate figures and perform meta-analyses. We reported estimated effect sizes with corresponding 95% confidence intervals (95% CI). A *p* < 0.05 was considered statistically significant.

## Results

### Demographical Data of the Studies Included

**Figure 1:**
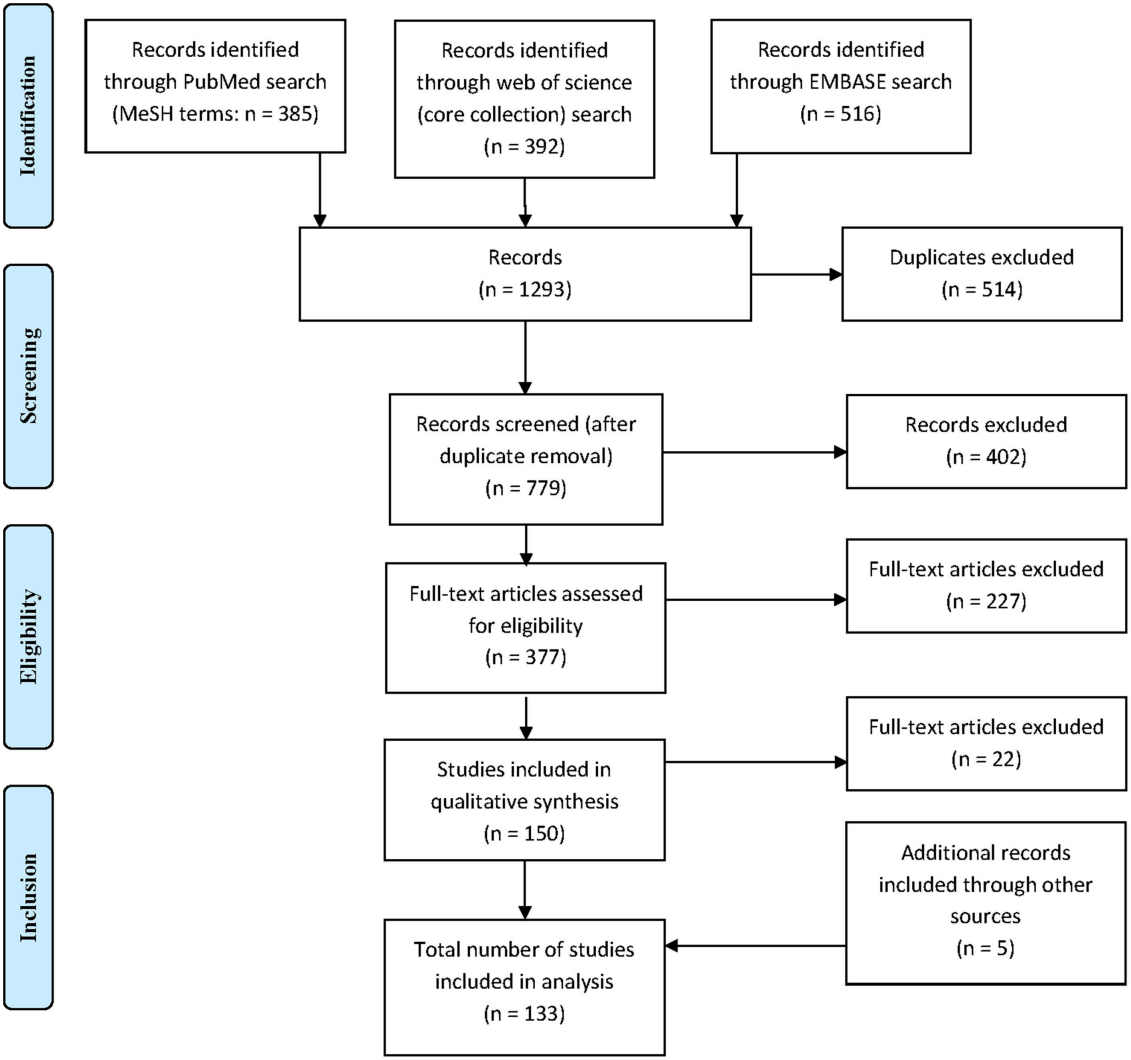
Flow chart outlining selection of articles included in the review.

A total of 1,293 citations were identified, of which 379 publications were eligible for full-text assessment and 133 (5 of which were found through additional sources) were included in the final analysis (Figure 1). As 21 publications included data on both QuantiFERON-TB and T-SPOT.TB and one study included data on two different QuantiFERON-TB tests a total of 155 datasets were generated. Table 1 provides an overview of the studies included and summarizes their key characteristics.

**Table 1:** Study results and characteristics of all included papers, sorted by year of publication.

**Study number**	**Authors**	**Year of publication**	**Country**	**IGRA**	**Participants n**	**Positive results n**	**Negative results n**	**Indeterminate results n**	**Proportion of ind. %**	**Mean age years**	**Median age years**	**Age range years**	**Years study performed**	**BCG vaccinated n (%)**	**HIV positive n (%)**	**Other immunodeficiency**	**Other immunodeficiency (%)**
1	Connell et al. (23)	2006	Australia	QFT Gold	101	20	64	17	16.8%	ns	ns	0.4–17.9	2004–2005	52.5%	ns	ns	ns
2	Dogra et al. (24)	2007	India	QFT Gold	105	11	94	0	0%	ns	6	1–12	2004–2005	81.9%	ns	ns	ns
3a	Domínguez et al. (25)	2008	Spain	QFT-GIT	134	50	84	0	0%	ns%	ns	0–18	2004–2006	64.2%	0%	ns	ns
3b	Domínguez et al. (25)	2008	Spain	T-SPOT.TB	134	51	80	3	2.2%	ns%	ns	0–18	2004–2006	64.2%	0%	ns	ns
4	Taylor et al. (26)	2008	UK	QFT	120	6	107	7	5.8%	10%	ns	0.3–16	2004–2005	46.7%	ns	ns	ns
5	Okada et al. (27)	2008	Cambodia	QFT Gold	204	33	162	9	4.4%	ns%	ns	0–5	2005	80%	ns	ns	ns
6	Ruhwald et al. (28)	2008	Nigeria	QFT-GIT	120	48	53	19	15.8%	ns%	ns	0–15	2005	85.8%	ns	ns	ns
7a	Mandalakas et al. (29)	2008	South Africa	QFT Gold	12	2	10	0	0%	4.4%	ns	ns	2005–2006	ns	100%	ns	ns
7b	Mandalakas. (29)	2008	South Africa	T-SPOT.TB	23	12	11	0	0%	4.4%	ns	ns	2005–2006	91.3%	100%	ns	ns
8	Ohno et al. (30)	2008	Japan	QFT Gold	17	0	17	0	0%	ns%	ns	0–10	2006	35.3%	ns	ns	ns
9	Soysal et al. (31)	2008	Turkey	T-SPOT.TB	209	31	173	5	2.4%	8.4%	ns	6–10	2006	90%	ns	ns	ns
10	Chun et al. (32)	2008	South Korea	QFT-GIT	227	16	194	17	7.5%	ns%	3.2	0–15.7	2006–2007	99.6%	0%	ns	ns
11a	Connell et al. (33)	2008	Australia	QFT-GIT	96	28	65	3	3.1%	8.9%	ns	0.5–19	ns	52.1%	0%	ns	ns
11b	Connell et al. (33)	2008	Australia	T-SPOT.TB	96	25	57	14	14.6%	8.9%	ns	0.5–19	ns	52.1%	0%	ns	ns
12	Petrucci et al. (34)	2008	Nepal, Brasil	QFT-GIT	259	117	136	6	2.3%	8.5%	ns	0.2–15	ns	96.5%	ns	ns	ns
13a	Richeldi et al. (35)	2008	Italy	QFT Gold	70	9	51	10	14.3%	6.1%	ns	ns	ns	ns	ns	ns	ns
13b	Richeldi et al (35)	2008	Italy	QFT-GIT	81	8	63	10	12.3%	6.9%	ns	ns	ns	ns	ns	ns	ns
14	Bianchi et al. (36)	2009	Italy	QFT-GIT	336	60	274	2	0.6%	ns%	4.5	2.6–6.8	2005–2006	51.5%	ns	ns	ns
15a	Hesseling et al. (37)	2009	South Africa	QFT Gold	21	8	10	3	14.3%	2.9%	ns	0–5	2005–2006	100%	0%	ns	ns
15b	Hesseling et al. (37)	2009	South Africa	T-SPOT.TB	28	25	2	1	3.6%	2.9%	ns	0–5	2005–2006	100%	0%	ns	ns
16	Higuchi et al. (38)	2009	Japan	QFT	308	6	300	2	0.6%	9.2%	ns	8–12	2005–2006	99%	ns	ns	ns
17	Kobashi et al. (39)	2009	Japan	QFT-2G	25	20	2	3	12%	ns%	ns	0–19	2005–2008	52%	0%	Immunosuppressive treatment	4%
18a	Kampmann et al. (40)	2009	UK	QFT-GIT	209	80	115	14	6.7%	8%	ns	0.2–16	2006–2008	67.9%	ns	ns	ns
18b	Kampmann et al. (40)	2009	UK	T-SPOT.TB	206	70	118	18	8.7%	8%	ns	0.2–16	2006–2008	67.9%	ns	ns	ns
19	Haustein et al. (16)	2009	UK	QFT Gold	237	41	113	83	35%	ns%	7.3	0–18	2006–2008	50.6%	0.8%	Inflammatory disorder, organ transplantation, asplenia, malignancies	24.1%
20a	ruzzese et al. (41)	2009	Italy	QFT-GIT	80	1	63	16	20%	12.5%	ns	2–24	ns	0%	0%	Rheumatoid arthritis, liver transplantation, panarteritis	100%
20b	Bruzzese et al. (41)	2009	Italy	T-SPOT.TB	74	7	57	10	13.5%	12.5%	ns	2–24	ns	0%	0%	Rheumatoid arthritis, liver transplantation, panarteritis	100%
21	Lighter et al. (42)	2009	USA	QFT-GIT	207	31	173	3	1.4%	9%	ns	0.1–18	ns	35.7%	ns	ns	ns
22	Stavri et al. (43)	2009	Romania	QFT	36	17	10	9	25%	ns%	ns	12–18	ns	100%	100%	ns	ns
23a	Bamford et al. (44)	2010	UK	QFT-GIT	170	101	56	13	7.6%	8.5	ns	0.2–16	2005–2007	ns	ns	ns	ns
23b	Bamford et al. (44)	2010	UK	T-SPOT.TB	94	47	47	0	0%	8.5	ns	0.2–16	2005–2007	ns	ns	ns	ns
24	Soborg et al. (45)	2010	Greenland	QFT Gold	2117	197	1898	22	1%	11.4	ns	0–18	2005–2007	21.7%	ns	ns	ns
25	Grare et al. (46)	2010	France	QFT-GIT	51	5	39	7	13.7%	6	ns	0.3–18	2007–2008	41.2%	0%	ns	ns
26a	Lucas et al. (47)	2010	Australia	QFT-GIT	460	45	345	70	15.2%	ns	ns	0.4–16	2007–2008	70%	0%	Schistosomiasis, Malaria, Hepatitis, Strongyloides	ns
26b	Lucas et al. (47)	2010	Australia	T-SPOT.TB	420	38	374	8	1.9%	ns	ns	0.4–16	2007–2008	70%	0%	Schistosomiasis, Malaria, Hepatitis, Strongyloides	ns
27a	Stefan et al. (48)	2010	South Africa	QFT-GIT	34	3	26	5	14.7%	ns	7	0.2–15	2007–2008	100%	0%	Cancer	100%
27b	Stefan et al. (48)	2010	South Africa	T-SPOT.TB	27	6	17	4	14.8%	ns	7	0.2–15	2007–2008	100%	0%	Cancer	100%
28	Tsolia et al. (49)	2010	Greece	QFT-GIT	286	125	152	9	3.1%	ns	ns	0–15	2007–2008	ns	ns	ns	ns
29	Thomas et al. (20)	2010	Bangladesh	QFT-GIT	302	107	121	74	24.5%	13.1	ns	11–15.3	2009	79.1%	ns	Helminth infection and malnutrition	83.1%
30a	Adetifa et al. (50)	2010	Gambia	QFT-GIT	215	72	141	2	0.9%	ns	ns	0.5–14	ns	59.1%	1.4%	ns	ns
30b	Adetifa et al. (50)	2010	Gambia	T-SPOT.TB	215	71	144	0	0%	ns	ns	0.5–14	ns	59.1%	1.4%	ns	ns
31	Kabeer et al. (51)	2010	India	QFT-GIT	83	2	81	0	0%	ns	ns	0–15	ns	74.7%	0%	ns	ns
32	Stavri et al. (52)	2010	Romania	QFT Gold	60	27	15	18	30%	9.44	ns	1–18	ns	100%	ns	ns	ns
33a	Altet-Gómez, et al. (53)	2011	Spain	QFT-GIT	166	61	105	0	0%	9.1	ns	0–15	2005–2007	69.9%	ns	ns	ns
33b	Altet-Gómez et al. (53)	2011	Spain	T-SPOT.TB	166	64	99	3	1.8%	9.1	ns	0–15	2005–2007	69.9%	ns	ns	ns
34	Cruz et al. (54)	2011	USA	T-SPOT.TB	215	70	135	10	4.7%	8.6	ns	0.1–18	2005–2006	36.3%	0%	ns	ns
35	Moyo et al. (55)	2011	South Africa	QFT-GIT	397	68	308	21	5.3%	ns	1.9	0.7–2.9	2005–2008	100%	0.5%	ns	ns
36	Banach et al. (56)	2011	USA	QFT Gold	6629	290	6203	136	2.1%	ns	ns	0–19	2006–2008	ns	ns	ns	ns
37	Kasambira et al. (57)	2011	South Africa	QFT-GIT	270	79	172	19	7%	ns	6	0.5–16	2006–2009	95.2%	5.2%	ns	ns
38	Losi et al. (58)	2011	Italy	QFT-GIT	235	80	152	3	1.3%	ns	ns	ns	2006–2008	76.6%	ns	ns	ns
39	Shah et al. (59)	2011	South Africa	QFT-GIT	196	62	117	17	8.7%	6	ns	0.5–16	2006–2009	94.9%	3.6%	ns	ns
40	Maritsi et al (60)	2011	UK	QFT-GIT	23	1	20	2	8.7%	ns	8.9	1.5–13	2007	21.7%	ns	Autoimmune disease	100%
41	Thomas et al. (61)	2011	UK	QFT-GIT	283	29	236	18	6.4%	5.3	ns	0–16	2007-2009	71.7%	ns	ns	ns
42	Zrinski et al. (62)	2011	Croatia	QFT-GIT	2173	485	1678	10	0.5%	ns	ns	0.1–18	2007–2010	100%	ns	ns	ns
43	Debord et al. (63)	2011	France	QFT-GIT	19	15	4	0	0%	ns	1.52	0.3–5.4	2008–2010	84.2%	0%	ns	ns
44	Pavić et al. (64)	2011	Croatia	QFT Gold	142	18	123	1	0.7%	2.4	ns	0.1–5	2008–2009	100%	ns	ns	ns
45	Mount et al. (65)	2011	UK	QFT Gold	126	92	29	5	4%	6.2	ns	0.2–16.4	2009–2011	99%	ns	ns	ns
46	Borgia et al. (66)	2011	Italy	QFT GIT	1340	118	1219	3	0.2%	ns	ns	0–0.25	2011	ns	ns	ns	ns
47	Yassin et al. (67)	2011	Ethiopia	QFT-GIT	737	256	308	173	23.5%	ns	ns	1–15	Ns	72.5%	7.1%	ns	ns
48	Buonsenso et al. (68)	2012	Italy	QFT Gold	66	64	1	1	1.5%	ns	ns	0–16	1990–2009	ns	3%	ns	ns
49	Riazi et al. (69)	2012	USA	QFT-GIT	517	27	453	37	7.2%	ns	ns	0.1–18	2004–2011	68.7%	ns	ns	ns
50	Banfield et al. (70)	2012	Australia	QFT Gold, QFT-GIT	573	57	423	93	16.2%	ns	ns	0–17	2006–2007	ns	0%	Helminth infection	40%
51a	Basu Roy et al. (71)	2012	Bulgaria, Greece, Italy, Spain, UK	QFT-GIT	1093	331	742	20	1.8%	8.2	ns	0–16	2006–2009	61.7%	ns	ns	ns
51b	Basu Roy et al. (71)	2012	Bulgaria, Greece, Italy, Spain, UK	T-SPOT.TB	382	145	231	6	1.6%	8.2	ns	0–16	2006–2009	61.7%	ns	ns	ns
52	Critselis et al. (72)	2012	Greece	QFT-GIT	761	221	517	23	3%	7.84	ns	0–18	2007–2010	45.2%	ns	ns	ns
53	Mendez-Echevarria et al. (73)	2012	Spain	QFT-GIT	459	96	343	20	4.4%	4.73	ns	0.1–15	2007–2009	46.4%	ns	ns	ns
54	Pong et al. (74)	2012	USA	QFT-GIT	23	22	0	1	4.3%	8.5	ns	0–16	2007–2010	ns	ns	ns	ns
55	Nenadic et al. (75)	2012	Croatia	QFT-GIT	59	57	2	0	0%	12	ns	4–18	2008–2009	100%	ns	ns	ns
56	Onur et al. (76)	2012	Turkey	QFT-GIT	97	37	54	6	6.2%	ns	ns	0.2–14	2008–2009	87.6%	ns	ns	ns
57	Rose et al. (77)	2012	Tanzania	QFT	211	26	128	57	27%	ns	ns	0–15	2008–2010	91%	37%	ns	ns
58	Kabeer et al. (78)	2012	India	QFT-GIT	145	32	113	0	0%	ns	ns	0–17	2008–2009	ns	ns	ns	ns
59	Tuuminen et al. (79)	2012	Finland	QFT-GIT	59	2	56	1	1.7%	ns	12	11–14	2008	ns	ns	ns	ns
60	Ling et al. (80)	2012	Canada	QFT-GIT	399	82	311	6	1.5%	ns	13	0–18	2009–2011	82%	ns	ns	ns
61	Nkurunungi et al. (81)	2012	Uganda	T-SPOT.TB	907	88	770	49	5.4%	5	ns	5	2009–2011	100%	1.4%	Helminth infection	9%
62	Verhagen et al. (82)	2012	Venezuela	QFT-GIT	140	48	80	12	8.6%	8.15	ns	1–15	2010–2011	86.4%	ns	Parasitic infection	97.1%
63	Dayal et al. (83)	2012	India	QFT-GIT	150	64	57	29	19.3%	ns	ns	0–18	ns	52%	ns	ns	ns
64	Rutherford et al. (84)	2012	Indonesia	QFT-GIT	371	171	190	10	2.7%	ns	5.1	0.2–10	ns	73.3%	ns	ns	ns
65	Blandinières et al. (85)	2013	France	QFT-GIT	226	53	150	23	10.2%	ns	ns	0–15	2007–2011	31.9%	ns	ns	ns
66a	Mandalakas et al. (86)	2013	South Africa	QFT-GIT	238	57	171	10	4.2%	ns	3.25	0.2–14.6	2007-2010	93%	50.8%	ns	ns
66b	Mandalakas et al. (86)	2013	South Africa	T-SPOT.TB	228	47	180	1	0.4%	ns	3.25	0.2–14.6	2007–2010	93%	50%	ns	ns
67	Yassin et al. (87)	2013	Ethiopia	QFT-GIT	458	158	223	77	16.8%	ns	ns	1–15	2007–2009	75.8%	5.9%	ns	ns
68	Chegou et al. (88)	2013	South Africa	QFT-GIT	76	41	33	2	2.6%	3.1	ns	0–13	2008	90%	28.9%	ns	ns
69	Rose et al. (89)	2013	Tanzania	QFT-GIT	152	20	93	39	25.7%	4.2	ns	0–15	2008–2010	95.4%	35.5%	ns	ns
70	Bua et al. (90)	2013	Italy	QFT-GIT	105	21	84	0	0%	ns	ns	0.2–15	2009–2011	1.9%	0%	ns	ns
71a	Carvalho et al. (91)	2013	Italy	QFT-GIT	18	0	15	3	16.7%	ns	5.5	1–18	2009–2010	ns	0%	Cancer	100%
71b	Carvalho et al. (91)	2013	Italy	T-SPOT.TB	17	2	12	3	17.6%	ns	6	1–18	2009-2010	ns	0%	Cancer	100%
72	Ling et al. (92)	2013	South Africa	T-SPOT.TB	557	175	353	29	5.2%	ns	1.9	0–15	2009-2011	80.3%	22.3%	ns	ns
73	Uluk et al. (93)	2013	Papua New Guinea	QFT-GIT	199	68	122	9	4.5%	ns	ns	0.1–12	2009–2010	75%	12.5%	ns	ns
74	Wassie et al. (94)	2013	Ethiopia	QFT-GIT	245	51	187	7	2.9%	14.8	15	12–20	2009	100%	0%	Helminth infection	20%
75	Laniado-Laborin et al. (95)	2013	Mexico	QFT-GIT	173	71	101	1	0.6%	7.6	ns	0–16	2011–2013	95.3%	ns	ns	ns
76	Dhanasekaran et al. (96)	2013	India	QFT-GIT	210	40	166	4	1.9%	ns	ns	0–3	ns	100%	ns	ns	ns
77	Lodha et al. (97)	2013	India	QFT-GIT	362	297	58	7	1.9%	ns	9.6	0.5–15	ns	74%	0%	ns	ns
78	Cranmer et al. (98)	2014	Kenya	T-SPOT.TB	160	14	114	32	20%	ns	ns	0–0.5	1999–2002	100%	7%	ns	ns
79	Garazzino et al. (99)	2014	Italy	QFT-GIT	823	126	662	35	4.3%	1.1	1.1	0–2	2005–2012	26.5%	ns	ns	ns
80	Hermansen et al. (100)	2014	Denmark	QFT-GIT	28	26	1	1	3.6%	ns	ns	1–14	2005–2010	ns	ns	ns	ns
81	Jenum et al. (101)	2014	India	QFT-GIT	691	36	633	22	3.2%	1.2	ns	0.1–2.9	2007–2010	100%	ns	ns	ns
82	Holm et al. (102)	2014	Tanzania	QFT-GIT	203	26	124	53	26.1%	ns	3	0–15	2008–2010	ns	37.4%	ns	ns
83	Song et al. (103)	2014	South Korea	QFT-GIT	2982	317	2649	16	0.5%	15.1	ns	11–19	2008–2012	61%	ns	ns	ns
84	Vallada et al. (104)	2014	Brasil	QFT-GIT	195	10	179	6	3.1%	3.9	ns	0.2–5.9	2008	100%	ns	ns	ns
85	Pérez-Porcuna et al. (105)	2014	Brasil	QFT-GIT	135	36	80	19	14.1%	ns	3.8	0–6	2009–2010	87.4%	ns	Helminth infection	22.2%
86a	Tieu et al. (106)	2014	Thailand	QFT-GIT	157	51	106	0	0%	7.2	ns	0.2–16	2009-2011	97.5%	1.9%	ns	ns
86b	Tieu et al. (106)	2014	Thailand	T-SPOT.TB	157	47	110	0	0%	7.2	ns	0.2–16	2009–2011	97.5%	1.9%	ns	ns
87	Bui et al. (107)	2014	USA	QFT-GIT	183	12	115	56	30.6%	11	ns	0–18	2010–2011	ns	15.8%	Cancer, autoimmune disease, inflammatory bowel disease	41%
88a	Chiappini et al. (108)	2014	Italy	QFT-GIT	332	96	236	0	0%	ns	5.5	ns	2010–2013	33%	ns	ns	ns
88b	Chiappini et al. (108)	2014	Italy	T-SPOT.TB	313	70	234	9	2.9%	ns	5.5	ns	2010–2013	33%	ns	ns	ns
89	Rose et al. (109)	2014	Canada	QFT-GIT	81	15	65	1	1.2%	12.5	ns	0-18	2010–2011	32.1%	100%	ns	ns
90	Verhagen et al. (110)	2014	Venezuela	QFT-GIT	151	63	77	11	7.3%	7.7	ns	0-16	2010–2011	86.8%	0%	ns	ns
91	Tebruegge et al. (15)	2014	UK	QFT-GIT	263	ns	ns	24	9.1%	ns	ns	0-18	2011–2013	ns	ns	Immunosuppressive therapy	3.1%
92	Al Mekaini et al. (111)	2014	Abu Dhabi	QFT-GIT	666	4	660	2	0.3%	ns	8.7	1–19	2013	71.6%	ns	ns	ns
93a	de Souza-Galvao et al. (112)	2014	Spain	QFT-GIT	37	23	14	0	0%	9.2	ns	ns	ns	67.6%	0%	ns	ns
93b	de Souza-Galvao et al. (112)	2014	Spain	T-SPOT.TB	37	21	16	0	0%	9.2	ns	ns	ns	67.6%	0%	ns	ns
94	Calzada-Hernandez et al. (113)	2015	Spain	QFT-GIT	75	3	66	6	8%	ns	ns	0–18	2004–2013	ns	ns	Autoimmune disease	100%
95	Caliman-Sturdza et al. (114)	2015	Romania	QFT-GIT	125	52	64	9	7.2%	10.45	ns	0.7–17	2006–2010	64.8%	12.8%	Diabetes, leukemia	2.4%
96	Sali et al. (115)	2015	Italy	QFT-GIT	621	59	536	26	4.2%	4.1	ns	0–14	2007–2010	ns	0.2%	Leukemia, juvenile arthritis, Evan's syndrome	1%
97a	Mandalakas et al. (116)	2015	South Africa	QFT-GIT	1295	520	741	34	2.6%	ns	4.9	0.2–15	2008–2012	86.7%	22%	ns	ns
97b	Mandalakas et al. (116)	2015	South Africa	T-SPOT.TB	1243	302	939	2	0.2%	ns	4.9	0.2–15	2008–2012	86.7%	21.4%	ns	ns
98	Spicer et al. (117)	2015	USA	T-SPOT.TB	107	5	91	11	10.3%	3.3	1.9	0.3–16	2008–2011	73.8%	0%	ns	ns
99	Bao et al. (118)	2015	China	QFT-GIT	57	28	28	1	1.8%	4.3	ns	0–16	2010–2011	ns	0%	Patients on glucocorticoid therapy	21.1%
100	Howley et al. (119)	2015	USA	QFT-GIT	2520	142	2365	13	0.5%	ns	ns	2–14	2010–2011	ns	ns	ns	ns
101	Pavic et al. (120)	2015	Croatia	QFT-GIT	171	26	143	2	1.2%	2.4	ns	0.1–5	2010–2012	98.8%	ns	ns	ns
102	Tebruegge et al. (121)	2015	Australia	QFT-GIT	142	22	103	2	1.4%	ns	8.3	0–18	2010–2011	47.2%	ns	ns	ns
103	Lebina et al. (122)	2015	South Africa	QFT-GIT	2105	351	1744	10	0.5%	ns	ns	5–7	2011	ns	ns	ns	ns
104	Petrone et al. (123)	2015	Uganda	QFT-GIT	105	17	81	7	6.7%	ns	ns	0–16	2011–2012	ns	29.5%	ns	ns
105	Sun et al. (124)	2015	China	T-SPOT.TB	579	119	411	49	8.5%	ns	ns	0–5	2011–2014	91%	0%	ns	ns
106a	Li et al. (125)	2015	China	QFT-GIT	57	28	28	1	1.8%	ns	ns	ns	ns	100%	0%	ns	ns
106b	Li et al. (125)	2015	China	T-SPOT.TB	96	46	50	0	0%	ns	ns	ns	ns	100%	0%	ns	ns
107	Cruz et al. (126)	2015	Botswana	QFT Gold	100	1	96	3	3%	ns	10.2	0.8–17	ns	92%	100%	ns	ns
108	Grinsdale et al. (127)	2016	USA	QFT-GIT, QFT Gold	1092	72	943	77	7.1%	ns	8.7	0–15	2005–2008	ns	ns	ns	ns
109	Santiago-Garcia et al. (128)	2016	Spain	QFT-GIT	81	64	11	6	7.4%	ns	ns	0–18	2005–2013	ns	ns	ns	ns
110	Perez-Porcuna et al. (129)	2016	Brasil	QFT	121	34	71	16	13.2%	ns	ns	0–6	2009–2010	100%	ns	ns	ns
111	Atikan et al. (130)	2016	Turkey	QFT-GIT	71	5	65	1	1.4%	8	ns	3.5–18	2010–2013	97.2%	ns	Rheumatic disease	100%
112	Boddu et al. (131)	2016	India	QFT-GIT	89	21	62	6	6.7%	ns	ns	1–15	2010–2011	98.9%	ns	ns	ns
113a	Nozawa et al. (132)	2016	Japan	QFT-GIT	81	4	69	8	9.9%	10.5	ns	1.1–19.2	2010–2014	95.1%	ns	Rheumatic disease	100%
113b	Nozawa et al. (132)	2016	Japan	T-SPOT.TB	27	0	27	0	0%	10.15	ns	3.3–19.8	2010–2014	96.3%	ns	Rheumatic disease	100%
114	El Azbaoui et al. (133)	2016	Morocco	QFT-GIT	109	40	49	20	18.3%	7.8	ns	0.4–17	2011–2015	100%	0%	ns	ns
115	Yun et al. (134)	2016	South Korea	QFT-GIT	106	15	88	3	2.8%	ns	9	0–18	2011–2015	99%	ns	ns	ns
116	Beshir et al. (135)	2016	Egypt	QFT-GIT	150	5	142	3	2%	1.4	0.75	0–12	2014–2015	82%	ns	ns	ns
117	Wong et al. (136)	2017	Taiwan	QFT-GIT	47	8	36	3	6.4%	10.2	ns	0.2–18	2008–2014	100%	ns	Leukemia	12.8%
118	Gabriele et al. (137)	2017	Greece	QFT-GIT	79	3	74	2	2.5%	ns	12	ns	2011–2012	30.4%	ns	Juvenile arthritis, lupus	100%
119	Mensah et al. (138)	2017	Ghana	QFT-GIT	32	20	10	2	6.3%	ns	ns	0–15	2012–2014	78.1%	ns	ns	ns
120	Li et al. (139)	2017	China	QFT-GIT	2831	71	2698	62	2.2%	9.6	ns	5–15	2013	64.2%	0%	ns	ns
121	Petrucci et al. (140)	2017	Italy	QFT-GIT	517	79	418	20	3.9%	5.4	ns	0–14	Ns	9.7%	ns	ns	ns
122	Silveira et al. (141)	2018	Brasil	T-SPOT.TB	86	21	52	13	15.1%	ns	9.8	0–19	2007–2011	83.7%	16.3%	Autoimmune disease, neoplasia, other immunodeficiencies	36.1%
123	Bielecka et al. (142)	2018	Poland	QFT-GIT	146	17	126	3	2.1%	ns	7.8	0–17	2009–2012	99%	0%	Juvenile arthritis, ulcerative colitis	1.4%
124	Chiappini et al. (143)	2018	Italy	QFT-GIT	762	32	730	0	0%	ns	3.6	0–18	2009–2015	53.9%	0.1%	Parasitic infection	53.7%
125	Mastrolia et al. (144)	2018	Italy	QFT-GIT	1779	86	1689	4	0.2%	ns	5.8	0–18	2009–2017	75.8%	0%	ns	ns
126	Mandalakas et al. (145)	2018	USA, Puerto Rico	T-SPOT.TB	43196	2189	40753	254	0.6%	ns	12.5	0–17	2010–2015	ns	ns	ns	ns
127	Gaensbauer et al. (146)	2018	USA	QFT	6336	450	5852	34	0.5%	ns	ns	2–18	2011–2014	ns	ns	ns	ns
128	Hormi et al. (147)	2018	France	QFT	63	8	51	4	6.3%	ns	11.6	0.4–18	2011–2015	92.1%	100%	ns	ns
129a	Starshinova et al. (148)	2018	Russia	QFT-GIT	312	201	111	0	0%	ns	ns	1–19	2011–2016	100%	0%	ns	ns
129b	Starshinova et al. (148)	2018	Russia	T-SPOT.TB	236	32	204	0	0%	ns	ns	1–19	2011–2016	100%	0%	ns	ns
130	Sayyahfar et al. (149)	2018	Iran	QFT	31	0	31	0	0%	8.79	ns	3–15	2013–2014	100%	ns	Renal dysfunction	100%
131	Said et al. (150)	2018	Tanzania	QFT Gold	301	39	244	18	6%	ns	2.2	0.5–4.9	2015–2016	100%	1.3%	Helminth infection	22.3%
132	Sali et al. (151)	2018	Italy	QFT	550	64	477	9	1.6%	5.8	ns	0–14	ns	43.5%	ns	ns	ns
133	Vortia et al. (152)	2018	USA	QFT-GIT	93	2	90	1	1.1%	ns	16	5–19	ns	ns	ns	Inflammatory bowel disease	100%
Total					107‘418			2555				0–24	1999–2018				

*QFT, QuantiFERON-TB, assay generation not specified; QFT-GIT, QuantiFERON-TB Gold in tube; ns, not specified*.

The 155 datasets included a total of 107,418 participants with a median number of participants of 166 (range 12–43,196) per dataset. The mean or median age was specified in 69% (107/155) of datasets and reported to be 7.6 and 6 years, respectively. Upper age range was 18 years in 87.2% (116/133), 19 years in 5.3% (7/133), 24 years in 2.3% (3/133), and not specified in 5.3% (7/133) of studies. The studies were done in 45 countries with 36.8% (49/133) in Europe, 21.8% (29/133) in Asia, 20.3% (27/133) in Africa, 11.3% (15/133) in North America, 4.5% (6/133) in Australia, 4.5% (6/133) in South America, and 0.75% (1/133) recruited children in two continents (Asia and South America).

The BCG vaccination rates were reported in 80% (124/155) of datasets and varied from 0 to 100% with a median of 82%. HIV infection rates were reported in 49% (76/155) and varied from 0 to 100% with the median infection rate of 0.05%.

In 33 datasets additional information on immunocompromising or other factors potentially influencing IGRA results was reported: rheumatic or autoimmune diseases in 12.3% (19/155), various forms of cancer in 4.5% (7/155), and parasitic infections in 6.5% (10/155) of datasets. The range of participants included with additional factors varied from 1 to 100% with a median of 83.1% (not specified in 2 datasets).

### Definition of Indeterminate Results of Interferon-Gamma Release Assays

A definition for indeterminate results was included in 88% (117/133) of studies with definitions provided for QuantiFERON-TB in 85.7% (108/126) and for T-SPOT.TB in 96.7% (29/30) of datasets. Of those that included a definition for indeterminate results most datasets 49.7% (77/155) simply stated to have used the manufacturers' definition [QuantiFERON-TB 47.6% (60/126) and T-SPOT.TB 56.7% (17/30)]. Further to this for the definition of indeterminate results in the QuantiFERON-TB assay three studies used their own definitions for failed nil controls [nil tube interferon-γ concentration of > 0.7 IU/ml (56) and > 2.0 IU/ml (63, 147), respectively]; five studies stated presence of high background response without reporting specific values (23, 36, 47, 70, 74).

Definition of indeterminate results for the T-SPOT.TB most commonly referred to low mitogen and/or high nil responses in combination with negative antigen response without stating specific values. Some studies indicated the absolute number of spots as cut-offs, others defined the number of spots in relation to the nil and/or mitogen response. In four studies a nil control of more than 10 spots was considered indeterminate, as opposed to the manufacturer's definition of ≥ 6 spots (92, 98, 112, 145).

### Type of Interferon-Gamma Release Assays

Of the 133 studies, 77.4% (103/133) assessed QFT only, 15.8% (21/133) assessed both QuantiFERON-TB and T-SPOT.TB, and 6.8% (9/133) assessed T-SPOT.TB only. The proportions of indeterminate results ranged from 0 to 35% in the included studies. The overall pooled effect size (equivalent to the pooled proportion of indeterminate results) was 0.04 (95% CI 0.03–0.05, I^2^ = 96.32%) for both IGRAs combined.

**Figure 2:**
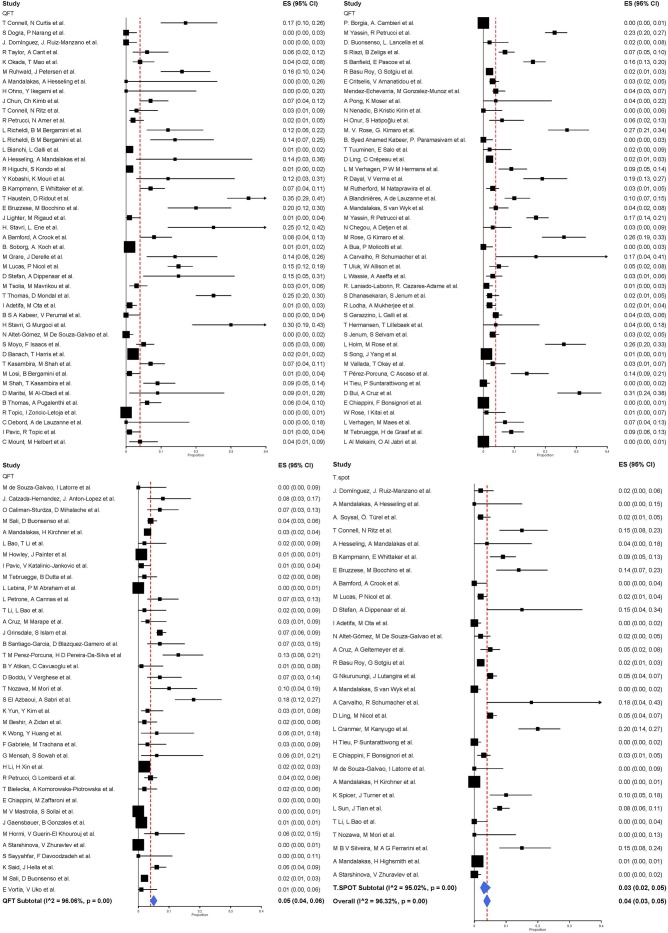
Proportion of indeterminate results with 95% CI by type of IGRA. Studies arranged according to year of publication.

QuantiFERON-TB was used in 124 studies including 57,183 participants. The pooled proportion of indeterminate results of QuantiFERON-TB was 0.05 (95% CI 0.04–0.06, I^2^ = 96.06%) (Figure 2). T-SPOT.TB was analyzed in 30 studies including 50,235 participants. The pooled proportion of indeterminate results of T-SPOT.TB was 0.03 (95% CI 0.02–0.05, I^2^ = 95.02%).

**Figure 3:**
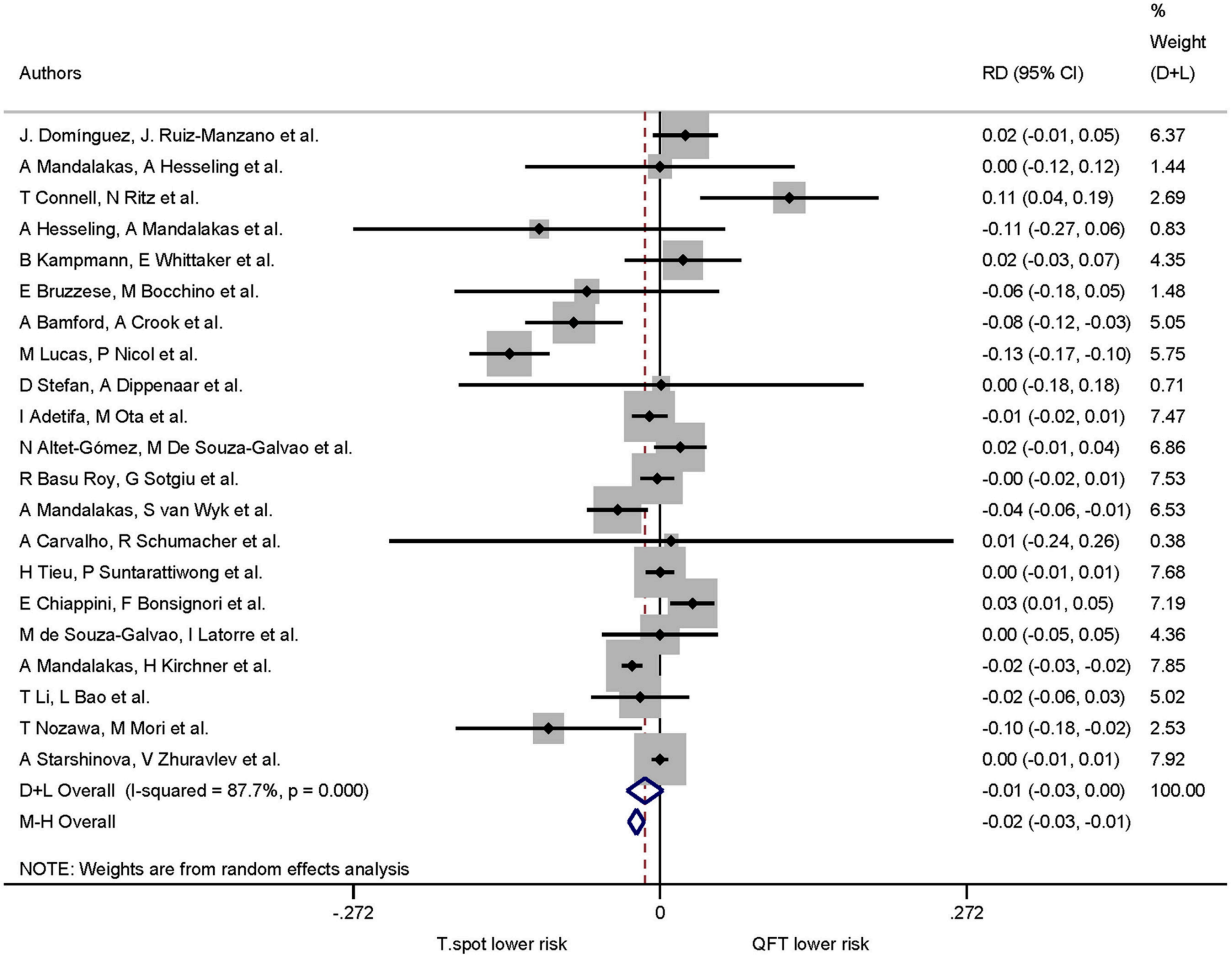
Risk difference (RD) with 95% CI in studies that included a head-to-head comparison of QuantiFERON-TB and T-SPOT.TB assays. Studies arranged according to year of publication.

A total of 21 studies assessed QuantiFERON-TB and T-SPOT.TB in the same study which allowed calculation of risk differences for the proportion of indeterminate results. The pooled proportion of indeterminate results was lower for T-SPOT.TB compared to QuantiFERON-TB (risk difference −0.01, 95% CI −0.03 to −0.00, I^2^ = 87.7%), but did not reach statistical significance (Figure 3).

### Indeterminate Results According to Age

The mean or median age was specified in 108 datasets; of those 55 datasets had median or mean ages 0–7 years 52 datasets had median or mean ages ≥8years. The pooled proportions of indeterminate results were 0.04 (95% CI 0.03–0.06, I^2^ = 94.46%) for the age group 0–7 years and 0.04 (95% CI 0.03–0.05, I^2^ = 95.19%) for ≥8years. For the 48 datasets in which the mean or median age was not specified the proportions of indeterminate results were 0.05 (95% CI 0.03–0.06, I^2^ = 97.35%).

**Figure 4:**
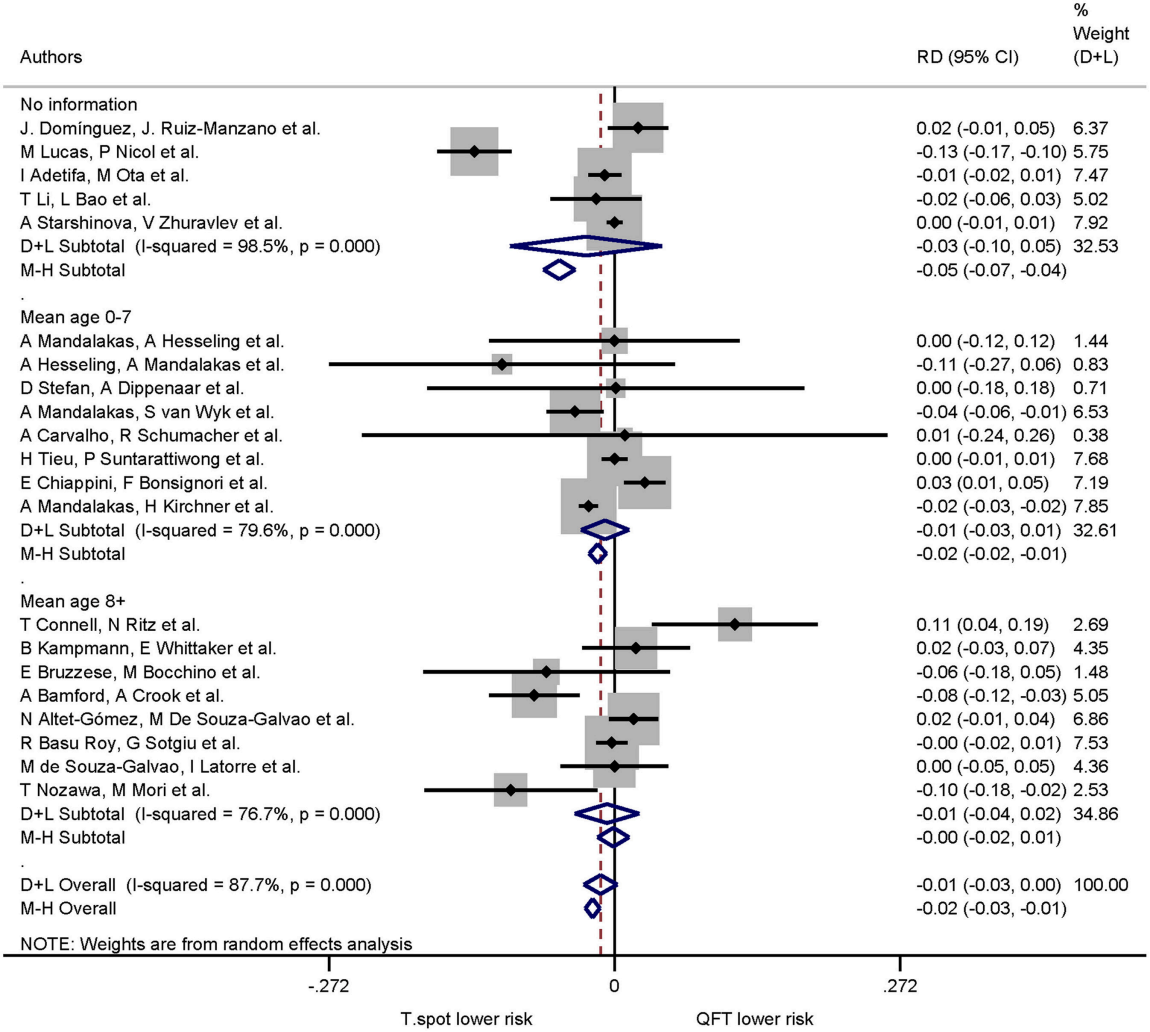
Risk differences (RD) with 95% CI in studies that included a head-to-head comparison of QuantiFERON-TB and T-SPOT.TB assays stratified by age. Studies sorted according to year of publication.

Of the 21 studies comparing both QuantiFERON-TB and T-SPOT.TB, 16 studies specified mean or median age. The pooled risk difference (negative values indicating lower risk of indeterminate results in the T-SPOT.TB) for 0–7 years was −0.01 (95% CI −0.03 to −0.01, I^2^ = 79.6%) and for ≥8 years −0.01 (95% CI −0.04 to −0.02, I^2^ = 76.7%). For studies which did not specify mean or median age the pooled risk difference was −0.03 (95% CI −0.10 to −0.05, I^2^ = 98.5%) (Figure 4). Risk differences within age groups for both assays were not statistically significant.

### Indeterminate Results According to Geographical Location of the Study Population

A stratified analysis according to continents showed the following proportions for indeterminate IGRA results: Europe 0.03 (95% CI 0.02–0.05, I^2^ = 93.49%), Africa 0.07 (95% CI 0.04–0.10, I^2^ = 97.02%), Australia 0.08 (95% CI 0.04–0.14, I^2^ = 94.33%), Asia 0.03 (95% CI 0.01–0.04, I^2^ = 93.12%), North America 0.03 (95% CI 0.02–0.05, I^2^ = 97.48%), South America 0.09 (95% CI 0.06–0.14, I^2^ = 77.03%). One report with study sites in Asia and South America was excluded from this particular analysis, as the data could not be separated according to site of recruitment (34).

**Figure 5:**
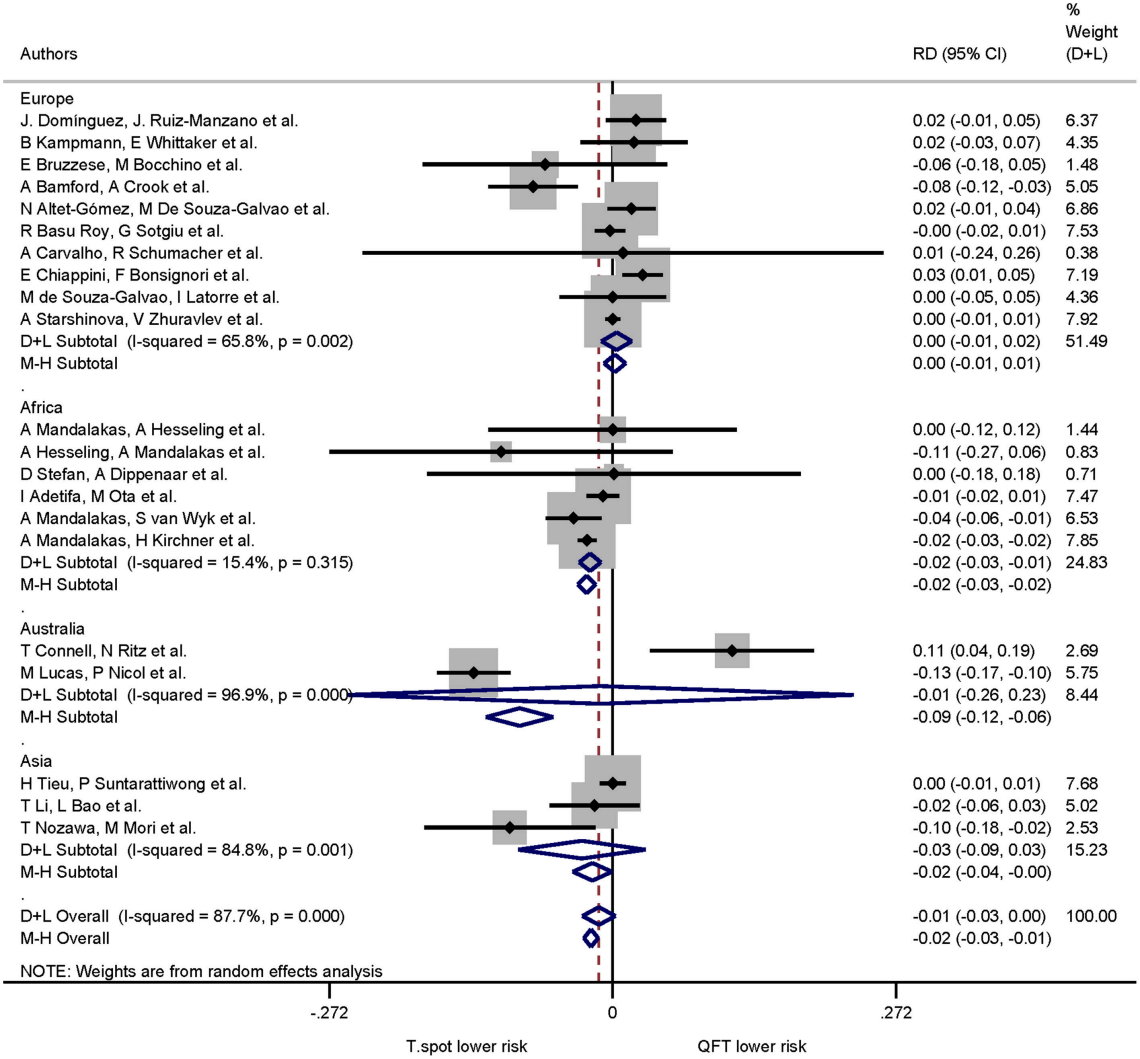
Risk differences (RD) with 95% CI in studies that included a head-to-head comparison of QuantiFERON-TB and T-SPOT.TB assays stratified by continent. Studies arranged according to year of publication.

**Figure 6:**
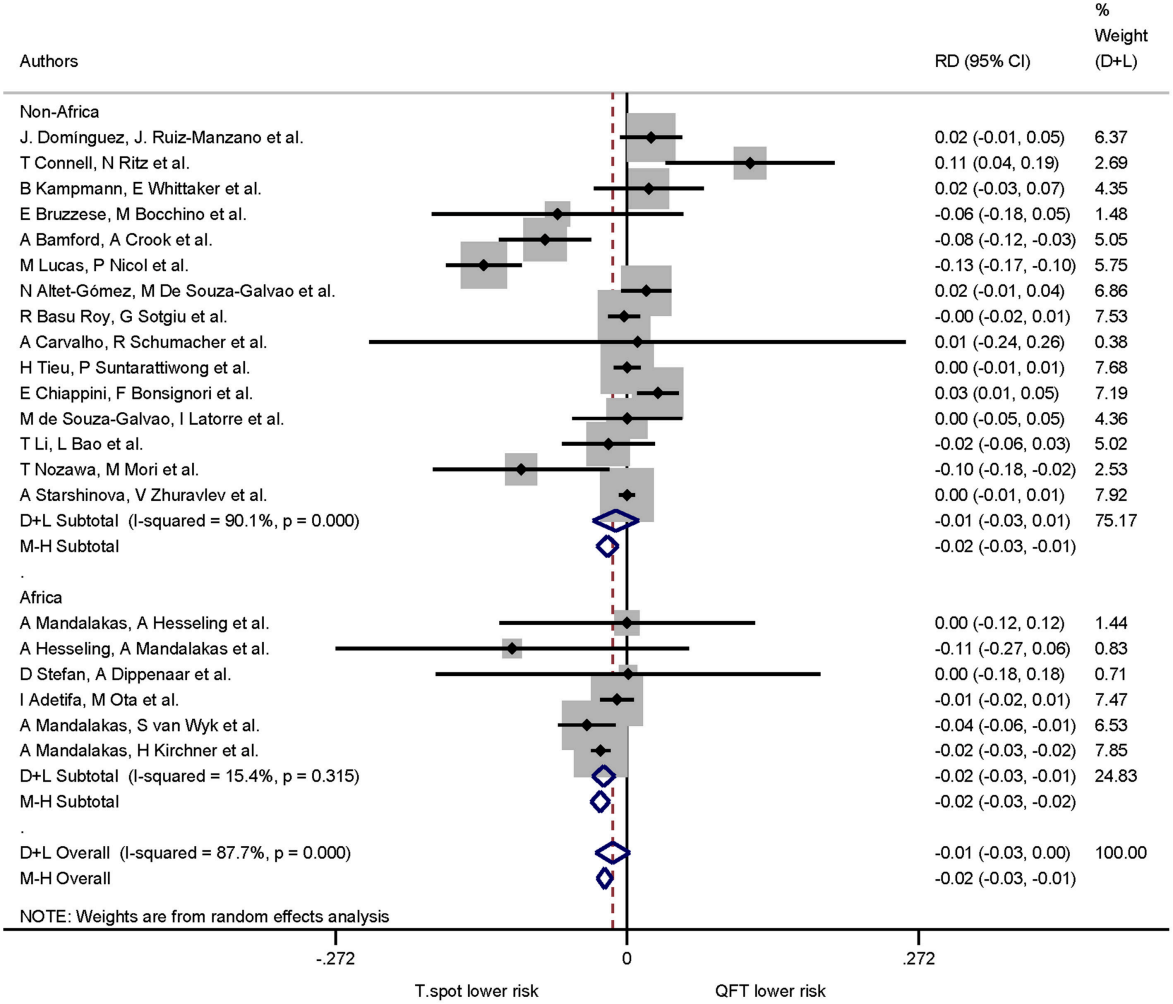
Risk differences (RD) with 95% CI in studies that included a head-to-head comparison of QuantiFERON-TB and T-SPOT.TB assays stratified by African/Non-African origin of the study. Studies arranged according to year of publication.

When continent of the study was included in the risk differences analysis the proportion of indeterminate results for T-SPOT.TB was significantly lower compared to QuantiFERON-TB in studies performed in Africa only (*p* < 0.001). The pooled risk difference for African studies was −0.022 (95% CI −0.032 to −0.011, I^2^ = 15.4%). Risk differences for studies performed on all other continents were not statistically significant (Figures 5, 6).

### Indeterminate Results by Immune Status

The pooled proportion of indeterminate IGRA results for the 0% HIV^+^, 0 < 51% HIV^+^, 51–100% HIV^+^, immunocompromised/HIV^−^ and no information were 0.03 (95% CI 0.02–0.05, I^2^ = 94.45%), 0.07 (95% CI 0.04–0.11, I^2^ = 97.70%), 0.03 (95% CI 0.01–0.05, I^2^ = 73.96%), 0.12 (95% CI 0.07–0.18, I^2^ = 47.12%), 0.03 (95% CI 0.03–0.04, I^2^ = 94.78%), respectively.

**Figure 7:**
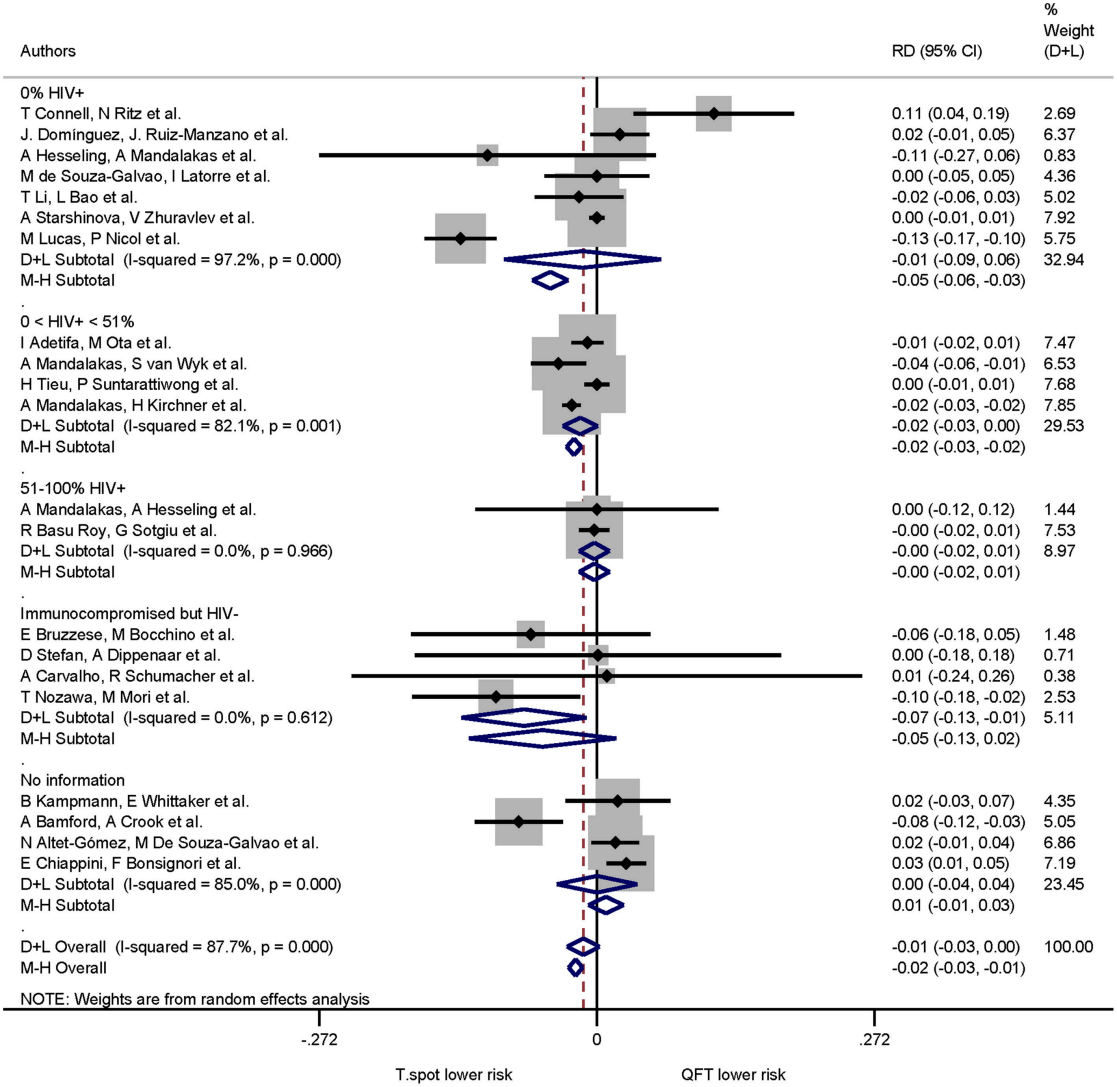
Risk differences (RD) with 95% CI in studies that included a head-to-head comparison of QuantiFERON-TB and T-SPOT.TB assays stratified by immune status. Studies arranged according to year of publication.

When immune status was included in the risk difference analysis of indeterminate results the T-SPOT.TB was associated with lower proportions of indeterminate results only in studies that included immunocompromised, HIV-uninfected participants: the pooled risk difference was −0.071(95% CI −0.133 to −0.010, I^2^ = 0.0%) and statistically significant (*p* = 0.022). The risk differences in the remaining groups were not statistically significant (Figure 7).

### Meta-Regression of Indeterminate Results

Of the four variables in the model (type of IGRA, age group, continent where study was performed, immune status), only studies including non-HIV-infected immunocompromised patients had a statistically significant contribution to the heterogeneity in the multiple regression model (*p* = 0.0003).

## Discussion

Indeterminate IGRA results have been reported shortly after introduction of these tests in routine clinical use. Despite this, analysis of indeterminate IGRA results has commonly been neglected in the literature, with those results either having been excluded from previous systematic literature reviews or only having been included in very limited subgroup analyses (11, 153, 154). To our knowledge, this systematic review is the first to comprehensively analyse the occurrence of indeterminate IGRA results in children and adolescents. We found that 4% of IGRA results are indeterminate, suggesting that 1 in 25 tests will not produce a conclusive result. The main factor associated with indeterminate results identified in this meta-analysis was the presence of an immunocompromising condition other than HIV infection.

In our analysis T-SPOT.TB assays were associated with a similar risk of indeterminate results compared to various generations of QuantiFERON-TB tests. T-SPOT.TB assays require lymphocyte adjustment which may reduce the risk of an indeterminate result particularly in patients with reduced lymphocyte count, such as HIV infection or immunocompromising conditions associated with lymphopenia. This assumption is confirmed by results from a meta-analysis including studies in adult HIV-infected patients showing that low CD4 cell counts increased indeterminate rates of QuantiFERON-TB but not of T-SPOT.TB assays (155). Our results contrast with another earlier meta-analysis by Diel et al that reported fewer indeterminate results for QFT-GIT (2.1%) compared to T-SPOT.TB (3.8%) (154). The authors concluded that the more demanding laboratory work for the T-SPOT.TB was likely the reason for higher indeterminate rates. However, their analysis predominately included studies in adults, did not include random effects models, and only included studies published until 2009.

Immunocompromising conditions, including HIV infection, have been identified in earlier studies as a major contributing factor to indeterminate results (16). A study by Oni et al. showed that HIV infection in adults increased the risk of indeterminate results, either through low positive control responses or high interferon-γ background concentrations in the negative control (156). In another study by Mandalakas et al. indeterminate results were more frequent in children infected with HIV than in HIV-uninfected children (116). The previously reported lower sensitivity of QuantiFERON-TB assays in HIV-infected individuals may be linked to a higher rate of indeterminate results, as the difference between the assays was negligible in a study after exclusion of indeterminate results in one analysis (157). Diel et al. reported in their meta-analysis that the rates of indeterminate results for QuantiFERON-TB and T-SPOT.TB assays were higher in immunocompromised compared to immunocompetent individuals, with 6.1 and 4.4%, respectively (154).

Further factors have been shown to influence IGRA results, particularly chronic rheumatic or auto-inflammatory diseases (158, 159). IGRA performance depends on intact cellular Th1 responses. Helminth infections, which primarily induce Th2 responses, may alter cytokine production and thereby increase the rate of indeterminate results (20, 150, 160, 161).

Importantly, in our analysis younger age was not associated with indeterminate results, reflected in similar proportions of indeterminate IGRA results in all age groups. This conflicts with several studies that have reported a clear correlation between IGRA performance, proportions of indeterminate results and age (15, 16, 18, 27, 33, 158). It is well-established that young children have a maturing immune system that may result in diminished cytokine release (162, 163). The link between age and cytokine concentrations has also been shown in numerous studies in healthy children unrelated to TB diagnostics (162, 163). One potential reason for not detecting a significant association between age and indeterminate IGRA results in this meta-analysis is that aggregate data based on the reported mean/median ages rather than individual patient data were used for this analysis.

There were several factors we were unable to analyse in the datasets that have been reported in some of the included studies, which mainly concern pre-analytical factors. Several studies in children and in adults found a decrease in interferon-γ production and indeterminate IGRA results to be associated with delayed sample incubation, shipping of samples, variation in environmental temperatures, and poor phlebotomy technique (164–168). In addition, co-medication may influence results as a recent *ex vivo* study showed that both corticosteroids and anti-TNF-alpha agents can cause false-negative IGRA results, and potentially also increase the rate of indeterminate results (169).

One potential limitation of our meta-analysis is the considerable heterogeneity of the included studies. Despite using empirical random effects weighting, excluding studies with < 10 participants, and using only data of the two commercially available IGRAs, heterogeneity remained. Moreover, it is possible that studies with poor IGRA performance and higher proportion of indeterminate results were less likely to be published, leading to publication bias. In addition, details on the type of QuantiFERON-TB assays used were often not reported in the publications, precluding a comparison of different test generations.

## Conclusions

In children, indeterminate IGRA results occur in 1 in 25 tests performed on average. Overall, there was no difference in the proportion of indeterminate results between both commercial assays. However, the data of this meta-analysis indicate that in patients in Africa and/or children with immunocompromising conditions other than HIV infection the T-SPOT.TB assay appears to produce fewer indeterminate results than the QuantiFERON-TB assays.

## Author Contributions

NR, MT, and UH conceptualized the study. NM and MG designed the search strategy and searched the literature, selected the studies and extracted the data. NR reviewed and approved the search strategy. NM, MG, and TV performed the data analysis. All authors performed the data interpretation. NM, MG, and NR wrote the draft manuscript. All authors reviewed, provided intellectual input into and approved the final manuscript.

### Conflict of Interest Statement

MT has received support from Cepheid for conference attendance. MT also received QuantiFERON-TB Gold assays at reduced cost for another research project from the manufacturer (Cellestis/Qiagen). The manufacturer had no influence on the study design, the data interpretation, the writing of the manuscript or the decision to submit the data for publication. The remaining authors declare that the research was conducted in the absence of any commercial or financial relationships that could be construed as a potential conflict of interest.

## Abbreviations

BCG: Bacille Calmette-Guérin
CI: Confidence interval
HIV: Human immunodeficiency virus
IGRA: Interferon-gamma release assay
IFN-γ: Interferon-gamma
ns: not specified
QFT: QuantiFERON-TB
TB: Tuberculosis
TST: Tuberculin skin test. ^*^QFT used representative for the two reported generations of QFT [QFT Gold and QFT-GIT (Gold In-Tube)].
