# Supplementary material for: Risk Factors for Indeterminate Interferon-Gamma Release Assay for the Diagnosis of Tuberculosis in Children—A Systematic Review and Meta-Analysis
Source: Front Pediatr. 2019 May 29;7:208. doi: 10.3389/fped.2019.00208 (PMC6548884; doi:10.3389/fped.2019.00208)
Supplement: Supplementary file 1 [file Data_Sheet_1.docx]

**Supplementary Material**

**File 1**: PRISMA checklist

| **Section/topic** | **#** | **Checklist item** | **Reported on page #** |
| --- | --- | --- | --- |
| **TITLE** | | |  |
| Title | 1 | Identify the report as a systematic review, meta-analysis, or both. | 1 |
| **ABSTRACT** | | |  |
| Structured summary | 2 | Provide a structured summary including, as applicable: background; objectives; data sources; study eligibility criteria, participants, and interventions; study appraisal and synthesis methods; results; limitations; conclusions and implications of key findings; systematic review registration number. | 1, 2 |
| **INTRODUCTION** | | |  |
| Rationale | 3 | Describe the rationale for the review in the context of what is already known. | 2 |
| Objectives | 4 | Provide an explicit statement of questions being addressed with reference to participants, interventions, comparisons, outcomes, and study design (PICOS). | 2 |
| **METHODS** | | |  |
| Protocol and registration | 5 | Indicate if a review protocol exists, if and where it can be accessed (e.g., Web address), and, if available, provide registration information including registration number. | 2, 3 |
| Eligibility criteria | 6 | Specify study characteristics (e.g., PICOS, length of follow-up) and report characteristics (e.g., years considered, language, publication status) used as criteria for eligibility, giving rationale. | 2, 3 |
| Information sources | 7 | Describe all information sources (e.g., databases with dates of coverage, contact with study authors to identify additional studies) in the search and date last searched. | 2, 3 |
| Search | 8 | Present full electronic search strategy for at least one database, including any limits used, such that it could be repeated. | suppl. |
| Study selection | 9 | State the process for selecting studies (i.e., screening, eligibility, included in systematic review, and, if applicable, included in the meta-analysis). | 2, Figure 1 |
| Data collection process | 10 | Describe method of data extraction from reports (e.g., piloted forms, independently, in duplicate) and any processes for obtaining and confirming data from investigators. | 2, 3 |
| Data items | 11 | List and define all variables for which data were sought (e.g., PICOS, funding sources) and any assumptions and simplifications made. | 3 |
| Risk of bias in individual studies | 12 | Describe methods used for assessing risk of bias of individual studies (including specification of whether this was done at the study or outcome level), and how this information is to be used in any data synthesis. | 3 |
| Summary measures | 13 | State the principal summary measures (e.g., risk ratio, difference in means). | 3 |
| Synthesis of results | 14 | Describe the methods of handling data and combining results of studies, if done, including measures of consistency (e.g., I^2^) for each meta-analysis. | 3 |

| **Section/topic** | **#** | **Checklist item** | **Reported on page #** |
| --- | --- | --- | --- |
| Risk of bias across studies | 15 | Specify any assessment of risk of bias that may affect the cumulative evidence (e.g., publication bias, selective reporting within studies). | 3 |
| Additional analyses | 16 | Describe methods of additional analyses (e.g., sensitivity or subgroup analyses, meta-regression), if done, indicating which were pre-specified. | 3 |
| **RESULTS** | | |  |
| Study selection | 17 | Give numbers of studies screened, assessed for eligibility, and included in the review, with reasons for exclusions at each stage, ideally with a flow diagram. | 9, Figure 1, suppl. |
| Study characteristics | 18 | For each study, present characteristics for which data were extracted (e.g., study size, PICOS, follow-up period) and provide the citations. | Table 1 |
| Risk of bias within studies | 19 | Present data on risk of bias of each study and, if available, any outcome level assessment (see item 12). | NA |
| Results of individual studies | 20 | For all outcomes considered (benefits or harms), present, for each study: (a) simple summary data for each intervention group (b) effect estimates and confidence intervals, ideally with a forest plot. | 9-13, Figures 2-7 |
| Synthesis of results | 21 | Present results of each meta-analysis done, including confidence intervals and measures of consistency. | 9-13 |
| Risk of bias across studies | 22 | Present results of any assessment of risk of bias across studies (see Item 15). | NA |
| Additional analysis | 23 | Give results of additional analyses, if done (e.g., sensitivity or subgroup analyses, meta-regression [see Item 16]). | 13 |
| **DISCUSSION** | | |  |
| Summary of evidence | 24 | Summarize the main findings including the strength of evidence for each main outcome; consider their relevance to key groups (e.g., healthcare providers, users, and policy makers). | 13-15 |
| Limitations | 25 | Discuss limitations at study and outcome level (e.g., risk of bias), and at review-level (e.g., incomplete retrieval of identified research, reporting bias). | 14, 15 |
| Conclusions | 26 | Provide a general interpretation of the results in the context of other evidence, and implications for future research. | 15 |
| **FUNDING** | | |  |
| Funding | 27 | Describe sources of funding for the systematic review and other support (e.g., supply of data); role of funders for the systematic review. | 15 |

**File 2:** Search strings for the three different databases

**PubMed, EMBASE**

*#A Search string for Tuberculosis:*

Tuberculosis OR TB

*#B Search string for IGRAs:*

((((t-spot.tb) OR t-spot) OR quantiferon-tb) OR quantiferon)

*#C Search string for children:*

Children OR pediatric OR paediatric

Search: #A AND #B AND #C

**Web of Science**

*#A Search string for Tuberculosis:*

TOPIC: (tubercu* OR TB)

*#B Search string for IGRAs:*

TOPIC: ((((t-spot.tb) OR t-spot) OR quantiferon-tb) OR quantiferon)

*#C Search string for children:*

TOPIC: child* OR pediatric OR paediatric

Search: #A AND #B AND #C

# File 3: Inclusion and exclusion criteria

Systematic review inclusion and exclusion criteria

I. Inclusion criteria

1. Study population
   1.1 Paediatric patient population up to an age range of 24 years
2. IGRA
   2.1 Inclusion of at least one commercially available IGRA to diagnose TB with reported numbers of positive, negative and indeterminate test results
3. Papers written in English, French or German

II. Exclusion criteria

1) Papers written in any language other than English, French or German

2) Reviews, case reports and case series

3) Studies with fewer than 10 participants

**File 4:** Reasons for exclusion of screened articles

| **Reason** | **Title / Abstract screen** | **Full-text screen** | **Data extraction** | **Total** |
| --- | --- | --- | --- | --- |
| **Duplicates** | 514 | - | - | 514 |
| **Case reports** | 91 | - | - | 91 |
| **Conference abstracts** | 89 | - | - | 89 |
| **Guidelines, recommendations** | 8 | - | - | 8 |
| **Letters, comments, books, editorials** | 15 | - | - | 15 |
| **Reviews, meta-analyses** | 96 | - | - | 96 |
| **Surveys of health practice** | 7 | - | - | 7 |
| **Results of different types of IGRAs mixed** | - | 1 | 3 | 4 |
| **Other outcomes (including other biomarkers etc.)** | 49 | 7 | - | 56 |
| **Language not English, French or German** | - | 23 | - | 23 |
| **No access** | - | 7 | - | 7 |
| **No commercial tests used** | - | 1 | 1 | 2 |
| **Adult or mixed population** | 47 | 100 | - | 147 |
| **No indeterminate results reported** | - | 82 | 14 | 96 |
| **Study population >10** | - | 3 | - | 3 |
| **Population unclear / exclusion of patients with indeterminate results** | - | 3 | - | 3 |
| **Same population as other study included in review** | - | - | 4 | 4 |
| **Total** | 916 | 227 | 22 | **1165** |

**File 5:** Non-exhaustive list of publications and their reasons for exclusion

| **Publication** | **Reason for exclusion** |
| --- | --- |
| Afzal, M. F., et al. (2017). "Latent tuberculosis infection (LTBI) in children less than 12 years of age in a tertiary care centre, Lahore, Pakistan." Pakistan Paediatric Journal 41(4): 207-210. | no indeterminate results reported |
| Altet, N., et al. (2015). "Predicting the Development of Tuberculosis with the Tuberculin Skin Test and QuantiFERON Testing." Ann Am Thorac Soc 12(5): 680-688. | no indeterminate results reported |
| Alsleben, N., et al. (2012). "Interferon-gamma inducible protein 10 as a biomarker for active tuberculosis and latent tuberculosis infection in children: A case-control study." Scandinavian Journal of Infectious Diseases 44(4): 256-262. | no indeterminate results reported |
| Andrews, J. R., et al. (2015). "The dynamics of QuantiFERON-TB gold in-tube conversion and reversion in a cohort of South African adolescents." Am J Respir Crit Care Med 191(5): 584-591. | no indeterminate results reported |
| Asl, H. M., et al. (2015). "QuantiFERON-TB Gold and Tuberculin Skin Test for the Diagnosis of Latent Tuberculosis Infection in Children." Iranian Journal of Medical Sciences 40(5): 411-417. | no indeterminate results reported |
| Bakir, M., et al. (2008). "Prognostic value of a T-cell-based, interferon-γ biomarker in children with tuberculosis contact." Annals of Internal Medicine 149(11): 777-786. | no indeterminate results reported |
| Balkhy, H. H., et al. (2016). "Comparison of QuantiFERON-TB gold in tube test versus tuberculin skin test for screening of latent tuberculosis infection in Saudi Arabia: A population-based study." Ann Thorac Med 11(3): 197-201. | no indeterminate results reported |
| Balkhy, H. H., et al. (2017). "Prevalence of Latent Mycobacterium Tuberculosis Infection (LTBI) in Saudi Arabia; Population based survey." Int J Infect Dis 60: 11-16. | no indeterminate results reported |
| Bergamini, B. M., et al. (2009). "Performance of Commercial Blood Tests for the Diagnosis of Latent Tuberculosis Infection in Children and Adolescents." Pediatrics 123(3): e419-e424. | IGRA test results mixed (with different cut-offs) |
| Biraro, I. A., et al. (2014). "Impact of co-infections and BCG immunisation on immune responses among household contacts of tuberculosis patients in a Ugandan cohort." Plos One 9(11): e111517. | no indeterminate results reported |
| Blount, R. J., et al. (2016). "Tuberculosis progression rates in U.S. Immigrants following screening with interferon-gamma release assays." Bmc Public Health 16(1): 875. | no indeterminate results reported |
| Çamlar, S. A., et al. (2011). "Performance of tuberculin skin test and interferon gamma assay for the diagnosis of latent tuberculosis infection in juvenile idiopathic arthritis." Clinical and Experimental Rheumatology 29(2): 386. | no indeterminate results reported |
| Chiappini, E., et al. (2012). "SERIAL T-SPOT.TB AND QUANTIFERON-TB-GOLD IN-TUBE ASSAYS TO MONITOR RESPONSE TO ANTITUBERCULAR TREATMENT IN ITALIAN CHILDREN WITH ACTIVE OR LATENT TUBERCULOSIS INFECTION." Pediatric Infectious Disease Journal 31(9): 974-977. | no indeterminate results reported |
| Clemente, M. G., et al. (2017). "Pediatric Tuberculosis in Northern Sardinia." Mediterranean Journal of Hematology and Infectious Diseases 9. | no indeterminate results reported |
| Cranmer, L. M., et al. (2018). "High Incidence of Tuberculosis Infection in HIV-exposed Children Exiting an Isoniazid Preventive Therapy Trial." Pediatr Infect Dis J 37(10): e254-e256. | no indeterminate results reported |
| Dehority, W., et al. (2017). "Comparison of the QuantiFERON TB Gold In-tube Assay With Tuberculin Skin Test for the Diagnosis of Latent Tuberculosis Infection Among HIV-infected and Uninfected Children." Pediatric Infectious Disease Journal 36(12): E317-E321. | no indeterminate results reported |
| Detjen, A. K., et al. (2007). "Interferon-γ release assays improve the diagnosis of tuberculosis and nontuberculous mycobacterial disease in children in a country with a low incidence of tuberculosis." Clinical Infectious Diseases 45(3): 322-328. | no indeterminate results reported |
| Dhanasekaran, S., et al. (2014). "Concordant or discordant results by the tuberculin skin test and the quantiFERON-TB test in children reflect immune biomarker profiles." Genes and Immunity 15(5): 265-274. | no indeterminate results reported |
| Dhanasekaran, S., et al. (2014). "Effect of Non-tuberculous Mycobacteria on Host Biomarkers Potentially Relevant for Tuberculosis Management." Plos Neglected Tropical Diseases 8(10). | no indeterminate results reported |
| Diel, R., et al. (2011). "Negative and positive predictive value of a whole-blood interferon-γ release assay for developing active tuberculosis: An update." American Journal of Respiratory and Critical Care Medicine 183(1): 88-95. | no indeterminate results reported |
| Díez, N., et al. (2015). "Use of interferon-gamma release assays to calculate the annual risk of tuberculosis infection." Pediatr Infect Dis J 34(2): 219-221. | no indeterminate results reported |
| Elliot, C., et al. (2018). "Tuberculin skin test versus interferon-gamma release assay in refugee children: A retrospective cohort study." Journal of Paediatrics and Child Health 54(8): 834-839. | no indeterminate results reported |
| Ferrarini, M. A. G., et al. (2016). "Rate of tuberculosis infection in children and adolescents with household contact with adults with active pulmonary tuberculosis as assessed by tuberculin skin test and interferon-gamma release assays." Epidemiology and Infection 144(4): 712-723. | no indeterminate results reported |
| Fireman-Klein, E., et al. (2015). "Interferon Gamma Release Assay-Guided Latent Tuberculosis Prophylaxis in Israel." Isr Med Assoc J 17(7): 405-409. | no indeterminate results reported |
| Gallant, C. J., et al. (2010). "Impact of age and sex on mycobacterial immunity in an area of high tuberculosis incidence." International Journal of Tuberculosis and Lung Disease 14(8): 952-959. | no indeterminate results reported |
| Gao, L., et al. (2017). "Incidence of active tuberculosis in individuals with latent tuberculosis infection in rural China: follow-up results of a population-based, multicentre, prospective cohort study." Lancet Infect Dis 17(10): 1053-1061. | no indeterminate results reported |
| Gonzalez-Salazar, F., et al. (2011). "Snapshot of Quantiferon TB gold testing in Northern Mexico." Tuberculosis 91: S34-S37. | no indeterminate results reported |
| Grare, M., et al. (2010). "Difficulties of TB diagnosis in children: QuantiFERON TB Gold (R) In-Tube as useful tool." Archives De Pediatrie 17(1): 77-85. | same test population as in other study already included in selection |
| Hanjiu, W., et al. (2013). "Application of the T-SPOT.TB assay to identify tuberculosis infection in children." Acta Medica Mediterranea 29(3): 443-446. | no indeterminate results reported |
| Hansted, E., et al. (2009). "T-cell-based diagnosis of tuberculosis infection in children in Lithuania: a country of high incidence despite a high coverage with bacille Calmette-Guerin vaccination." BMC Pulm Med 9: 41. | no indeterminate results reported |
| Herrmann, J. L., et al. (2009). "Temporal Dynamics of Interferon Gamma Responses in Children Evaluated for Tuberculosis." Plos One 4(1). | no indeterminate results reported |
| Higuchi, K., et al. (2008). "Relationship between whole-blood interferon-gamma responses and the risk of active tuberculosis." Tuberculosis (Edinb) 88(3): 244-248. | no indeterminate results reported |
| Higuchi, R., et al. (2009). "Whole blood interferon-γ assay for tuberculosis in children in Japan." Pediatrics International 51(1): 97-102. | no commercial IGRA used |
| Hu, Y., et al. (2013). "Prevalence of latent tuberculosis infection and its risk factors in schoolchildren and adolescents in Shanghai, China." European Journal of Public Health 23(6): 1064-1069. | no indeterminate results reported |
| Hur, Y. G., et al. (2014). "Identification of Immunological Biomarkers Which May Differentiate Latent Tuberculosis from Exposure to Environmental Nontuberculous Mycobacteria in Children." Clinical and Vaccine Immunology 21(2): 133-142. | no indeterminate results reported |
| Jablonka, A., et al. (2018). "Tuberculosis Specific Interferon-Gamma Production in a Current Refugee Cohort in Western Europe." Int J Environ Res Public Health 15(6). | no indeterminate results reported |
| Jacobs, S., et al. (2011). "The Tuberculin Skin Test Is Unreliable in School Children BCG-vaccinated in Infancy and at Low Risk of Tuberculosis Infection." Pediatric Infectious Disease Journal 30(9): 754-758. | no indeterminate results reported |
| Jeljeli, M., et al. (2017). "Cytokine/chemokine secretion for detecting tuberculosis in quantiferon supernatants from HIV+ and HIV− children." Journal of Infection 75(1): 77-80. | no indeterminate results reported |
| Jiang, B., et al. (2016). "Evaluation of interferon-gamma release assay (T-SPOT.TB (TM)) for diagnosis of tuberculosis infection in rheumatic disease patients." International Journal of Rheumatic Diseases 19(1): 38-42. | no indeterminate results reported |
| Kabeer, B. S. A., et al. (2012). "Interferon gamma and interferon gamma inducible protein-10 in detecting tuberculosis infection." Journal of Infection 64(6): 573-579. | no indeterminate results reported |
| Kampmann, B., et al. (2018). "Evaluating UK national guidance for screening of children for tuberculosis a prospective multicenter study." American Journal of Respiratory and Critical Care Medicine 197(8): 1058-1064. | IGRA test results mixed |
| Khalilzadeh, S., et al. (2010). "Role of quantiFERON-TB test in detection of children infected with mycobacterium tuberculosis." Tanaffos 9(3): 22-27. | no indeterminate results reported |
| Kim, H. J., et al. (2015). "Role of confirmatory interferon-gamma release assays in school outbreaks of tuberculosis in South Korea." Int J Tuberc Lung Dis 19(5): 576-581. | no indeterminate results reported |
| Komukai, J., et al. (2011). "[Comparative performance of tuberculin skin test and QuantiFERON TB-gold in contact investigations for tuberculosis]." Kekkaku 86(11): 847-856. | no indeterminate results reported |
| Krenke, K., et al. (2017). "Testing of tuberculosis infection among Chinese adolescents born after terminating the Bacillus Calmette-Guerin booster vaccination: subgroup analysis of a population-based cross-sectional study." Pediatr Pulmonol 11(4): 528-535. | same test population as in other study already included in selection |
| Lee, T., et al. (2010). "Diagnosis of latent tuberculosis infection by using the QuantiFERON-TB Gold in-tube test in children whose household contact has contagious pulmonary tuberculosis disease." International Journal of Infectious Diseases 14: E307-E307. | no indeterminate results reported |
| Leung, C. C., et al. (2015). "T-Spot.TB outperforms tuberculin skin test in predicting development of active tuberculosis among household contacts." Respirology 20(3): 496-503. | no indeterminate results reported |
| Li, R. L., et al. (2016). "Tuberculosis in infants: a retrospective study in China." Springerplus 5. | no indeterminate results reported |
| Lighter-Fisher, J., et al. (2010). "Cytokine responses to QuantiFERON® peptides, purified protein derivative and recombinant ESAT-6 in children with tuberculosis." International Journal of Tuberculosis and Lung Disease 14(12): 1548-1555. | no indeterminate results reported |
| Logan, E., et al. (2018). "Elevated IgG Responses in Infants Are Associated With Reduced Prevalence of Mycobacterium tuberculosis Infection." Frontiers in Immunology 9. | no indeterminate results reported |
| Lombardi, G., et al. (2018). "Quantitative Analysis of Gamma Interferon Release Assay Response in Children with Latent and Active Tuberculosis." Journal of Clinical Microbiology 56(2). | no indeterminate results reported |
| Lu, D., et al. (2016). "Diagnosis of Tuberculous Meningitis Using a Combination of Peripheral Blood T-SPOT.TB and Cerebrospinal Fluid Interferon-gamma Detection Methods." Laboratory Medicine 47(1): 6-12. | no indeterminate results reported |
| Lule, S. A., et al. (2015). "Factors associated with tuberculosis infection, and with anti-mycobacterial immune responses, among five year olds BCG-immunised at birth in Entebbe, Uganda." Vaccine 33(6): 796-804. | no indeterminate results reported |
| Machingaidze, S., et al. (2012). "Predictive value of recent QuantiFERON conversion for tuberculosis disease in adolescents." Am J Respir Crit Care Med 186(10): 1051-1056. | no indeterminate results reported |
| Mahmoudi, S., et al. (2017). "Interferon Gamma Release Assay in response to PE35/PPE68 proteins: a promising diagnostic method for diagnosis of latent tuberculosis." European Cytokine Network 28(1): 36-40. | no indeterminate results reported |
| Mahomed, H., et al. (2011). "The tuberculin skin test versus QuantiFERON TB Gold(R) in predicting tuberculosis disease in an adolescent cohort study in South Africa." Plos One 6(3): e17984. | no indeterminate results reported |
| Mahomed, H., et al. (2011). "Predictive factors for latent tuberculosis infection among adolescents in a high-burden area in South Africa." International Journal of Tuberculosis and Lung Disease 15(3): 331-336. | no indeterminate results reported |
| Mahomed, H., et al. (2013). "TB incidence in an adolescent cohort in South Africa." Plos One 8(3): e59652. | no indeterminate results reported |
| Mancuso, J. D., et al. (2011). "Impact of Targeted Testing for Latent Tuberculosis Infection Using Commercially Available Diagnostics." Clinical Infectious Diseases 53(3): 234-244. | no indeterminate results reported |
| Mandalakas, A. M., et al. (2012). "Well-quantified tuberculosis exposure is a reliable surrogate measure of tuberculosis infection." International Journal of Tuberculosis and Lung Disease 16(8): 1033-1039. | no indeterminate results reported |
| Markova, R., et al. (2011). "Association of age with the level of response in the QuantiFERON-TB Gold In-Tube assay for children with active tuberculosis." New Microbiologica 34(1): 81-85. | no indeterminate results reported |
| Marquez, C., et al. (2016). "Tuberculosis Infection in Early Childhood and the Association with HIV-exposure in HIV-uninfected Children in Rural Uganda." Pediatric Infectious Disease Journal 35(5): 524-529. | no indeterminate results reported |
| Masoumi Asl, H. M. M., et al. (2015). "QuantiFERON-TB Gold and Tuberculin Skin Test for the Diagnosis of Latent Tuberculosis Infection in Children." Iran J Med Sci 40(5): 411-417. | no indeterminate results reported |
| Méndez-Echevarría, A., et al. (2011). "Optimizing interpretation of the tuberculin test using an interferon-gamma release assay as a reference standard." Pediatric Infectious Disease Journal 30(5): 426-428. | same test population as in other study already included in selection |
| Molicotti, P., et al. (2008). "Performance of QuantiFERON-TB testing in a tuberculosis outbreak at a primary school." J Pediatr 152(4): 585-586. | no indeterminate results reported |
| Mukherjee, A., et al. (2014). "Effect of micronutrient deficiency on QuantiFERON-TB Gold In-Tube test and tuberculin skin test in diagnosis of childhood intrathoracic tuberculosis." European Journal of Clinical Nutrition 68(1): 38-42. | same test population as in other study already included in selection |
| Nakaoka, H., et al. (2006). "Risk for tuberculosis among children." Emerging Infectious Diseases 12(9): 1383-1388. | no indeterminate results reported |
| Neira-Munoz, E., et al. (2008). "Extensive transmission of mycobacterium tuberculosis among children on a school bus." Pediatric Infectious Disease Journal 27(9): 836-837. | no indeterminate results reported |
| Nejat, S. and R. Bennet (2016). "Interferon-gamma release assays can effectively screen migrants for the tuberculosis infection, but urgent, active cases need clinical recognition." Acta Paediatrica 105(6): 671-675. | IGRA test results mixed |
| Nicol, M. P., et al. (2009). "Comparison of T-SPOT.TB Assay and Tuberculin Skin Test for the Evaluation of Young Children at High Risk for Tuberculosis in a Community Setting." Pediatrics 123(1): 38-43. | no indeterminate results reported |
| Noorbakhsh, S., et al. (2011). "Evaluation of an interferon-gamma release assay in young contacts of active tuberculosis cases." East Mediterr Health J 17(9): 714-718. | no indeterminate results reported |
| Nsutebu, E., et al. (2008). "Use of QuantiFERON-TB Gold test in the investigation of unexplained positive tuberculin skin tests." Public Health 122(11): 1284-1287. | no indeterminate results reported |
| Perry, S., et al. (2008). "Reproducibility of QuantiFERON-TB gold in-tube assay." Clinical and Vaccine Immunology 15(3): 425-432. | no indeterminate results reported |
| Reechaipichitkul, W., et al. (2015). "Evaluation of the QuantiFERON?-TB Gold In-Tube assay and tuberculin skin test for the diagnosis of Mycobacterium tuberculosis infection in northeastern Thailand." Asian Pac J Allergy Immunol 33(3): 236-244. | no indeterminate results reported |
| Rego, K., et al. (2018). "Utility of the T-SPOT®.TB test's borderline category to increase test resolution for results around the cut-off point." Tuberculosis 108: 178-185. | no indeterminate results reported |
| Richeldi, L., et al. (2009). "Performance of Tests for Latent Tuberculosis in Different Groups of Immunocompromised Patients." Chest 136(1): 198-204. | no indeterminate results reported |
| Romagnoli, C., et al. (2012). "Neonatal tuberculosis: an experience that teaches." Journal of Maternal-Fetal & Neonatal Medicine 25: 38-41. | no indeterminate results reported |
| Rutherford, M. E., et al. (2012). "Risk factors for Mycobacterium tuberculosis infection in Indonesian children living with a sputum smear-positive case." International Journal of Tuberculosis and Lung Disease 16(12): 1594-1599. | no indeterminate results reported |
| Saraiva, R. and A. Dias (2013). "Prevalence of latent tuberculosis infection in a pediatric population in contact with high-risk adults." Atencion Primaria 45: 75. | no indeterminate results reported |
| Shimizu, H. and M. Mori (2017). "Usefulness of the Combination of Tuberculin Skin Test and Interferon-Gamma Release Assay in Diagnosing Children with Tuberculosis." Tohoku Journal of Experimental Medicine 243(3): 205-210. | no indeterminate results reported |
| Sollai, S., et al. (2017). "Infectious diseases prevalence, vaccination coverage, and diagnostic challenges in a population of internationally adopted children referred to a Tertiary Care Children's Hospital from 2009 to 2015." Medicine 96(12). | no indeterminate results reported |
| Stout, J. E., et al. (2018). "Evaluating latent tuberculosis infection diagnostics using latent class analysis." Thorax. | no indeterminate results reported |
| Sztajnbok, F., et al. (2014). "Tuberculin skin test and ELISPOT/T. SPOT.TB in children and adolescents with juvenile idiopathic arthritis." Pediatric Rheumatology 12. | no indeterminate results reported |
| Tavast, E., et al. (2009). "IGRA tests perform similarly to TST but cause no adverse reactions: pediatric experience in Finland." BMC Res Notes 2: 9. | no indeterminate results reported |
| Thomas, L., et al. (2016). "Is the QuantiFERON-TB Gold test (QFT) better than the Tuberculin Skin Test (TST) in diagnosing active and latent tuberculosis in BCG-vaccinated children?" International Journal of Infectious Diseases 45: 342-343. | no indeterminate results reported |
| Uzunhan, O., et al. (2015). "Comparison of tuberculin skin test and QuantiFERON (R)-TB Gold In-Tube for the diagnosis of childhood tuberculosis." Pediatrics International 57(5): 893-896. | no indeterminate results reported |
| Van der Zalm, M. M., et al. (2016). "The Effect of Deworming on Tests of Tuberculosis Infection in Children With Recent Tuberculosis Exposure: A Randomized Controlled Trial." Pediatr Infect Dis J 35(6): 622-627. | no indeterminate results reported |
| Walters, J. K. and A. D. Sullivan (2016). "Impact of Routine Quantiferon Testing on Latent Tuberculosis Diagnosis and Treatment in Refugees in Multnomah County, Oregon, November 2009-October 2012." J Immigr Minor Health 18(2): 292-300. | no indeterminate results reported |
| Wang, H. J., et al. (2013). "APPLICATION OF THE T-SPOT.TB ASSAY TO IDENTIFY TUBERCULOSIS INFECTION IN CHILDREN." Acta Medica Mediterranea 29(3): 443-446. | no indeterminate results reported |
| Wang, S. H., et al. (2010). "Evaluation of a modified interferon-gamma release assay for the diagnosis of latent tuberculosis infection in adult and paediatric populations that enables delayed processing." Scandinavian Journal of Infectious Diseases 42(11-12): 845-850. | no indeterminate results reported |
| Wang, X., et al. (2014). "The sensitivity of T-SPOT.TB assay in diagnosis of pediatric tuberculosis." Fetal Pediatr Pathol 33(2): 123-125. | no indeterminate results reported |
| Winje, B. A., et al. (2008). "School based screening for tuberculosis infection in Norway: Comparison of positive tuberculin skin test with interferon-gamma release assay." Bmc Infectious Diseases 8. | no indeterminate results reported |
| Yi, L. N., et al. (2016). "Evaluation of QuantiFERON-TB Gold Plus for Detection of Mycobacterium tuberculosis infection in Japan." Scientific Reports 6. | no indeterminate results reported |
| Yoo, R., et al. (2016). "Discordance between tuberculin skin test and interferon-gamma release assays for diagnosis of tuberculosis infection in Korean children." Pediatric Infection and Vaccine 23(1): 18-24. | no indeterminate results reported |
| Zhang, S., et al. (2010). "Evaluation of gamma interferon release assays using Mycobacterium tuberculosis antigens for diagnosis of latent and active tuberculosis in Mycobacterium bovis BCG-vaccinated populations." Clin Vaccine Immunol 17(12): 1985-1990. | no indeterminate results reported |
| Zhou, J. W., et al. (2014). "Comparison of the Interferon-Gamma Release Assay With the Traditional Methods for Detecting Mycobacterium tuberculosis Infection in Children." Medicine 93(15). | no indeterminate results reported |
